# A novel lncRNA-mediated signaling axis governs cancer stemness and splicing reprogramming in hepatocellular carcinoma with therapeutic potential

**DOI:** 10.1186/s13046-025-03546-w

**Published:** 2025-10-09

**Authors:** Ke Si, Lantian Zhang, Zehang Jiang, Zhiyong Wu, Zhanying Wu, Yubin Chen, Weifei Liang, Xiaoren Zhang, Wenliang Zhang

**Affiliations:** 1 The Key Laboratory of Advanced Interdisciplinary Studies, The First Affiliated Hospital of Guangzhou Medical University; GMU-GIBH Joint School of Life Sciences, The Guangdong-Hong Kong-Macao Joint Laboratory for Cell Fate Regulation and Diseases, Guangzhou Medical University, Guangzhou, 510182 China; 2 Affiliated Cancer Hospital and Institute of Guangzhou Medical University, Guangzhou, 510095 China; 3 Department of Clinical Oncology, The University of Hong Kong-Shenzhen Hospital, Shenzhen, 518053 Guangdong China; 4 KingMed School of Laboratory Medicine, Guangzhou Medical University, Guangzhou, 510182 China; 5 Department of Pediatrics, The University of Hong Kong-Shenzhen Hospital, Shenzhen, 518053 China

**Keywords:** LncRNA *RAB30-DT*, SRPK1, CDCA7, Alternative splicing, Cancer stem cell, Hepatocellular carcinoma

## Abstract

**Background:**

Aberrant alternative splicing (AS) contributes to cancer stemness and progression in hepatocellular carcinoma (HCC). However, the regulatory roles of long noncoding RNAs (lncRNAs) in linking AS dysregulation to tumor stemness remain elusive.

**Methods:**

We performed integrated bulk and single-cell RNA-Seq analyses combined with functional assays to identify key lncRNAs associated with splicing regulation and cancer stemness in HCC. Mechanistic studies were conducted to elucidate the molecular interplay between lncRNAs, splicing factors, and transcriptional regulators. Drug sensitivity assays were used to evaluate therapeutic potential.

**Results:**

Global analysis revealed increased splicing regulator activity during hepatocellular carcinoma (HCC) progression, which correlated with poor prognosis. This splicing dysregulation led us to identify 28 lncRNAs that connect aberrant splicing with cancer stemness. Among these, *RAB30-DT* was significantly overexpressed in malignant epithelial cells and associated with advanced tumor stage, stemness features, genomic instability, and poor patient prognosis. Functional assays demonstrated that *RAB30-DT* promotes proliferation, migration, invasion, colony and sphere formation in vitro, and tumor growth in vivo. Mechanistically, *RAB30-DT* is transcriptionally activated by CREB1 and directly binds and stabilizes the splicing kinase SRPK1, facilitating its nuclear localization. This interaction broadly reshapes the AS landscape, including splicing of the cell cycle regulator CDCA7, to drive tumor stemness and malignancy. Importantly, pharmacological disruption of the CREB1–RAB30-DT–SRPK1 axis sensitizes HCC cells to targeted therapies.

**Conclusions:**

Our study reveals a novel lncRNA-mediated signaling axis that integrates transcriptional regulation and splicing reprogramming to sustain cancer stemness and progression in HCC. Targeting this axis offers promising therapeutic opportunities for HCC treatment.

**Supplementary Information:**

The online version contains supplementary material available at 10.1186/s13046-025-03546-w.

## Graphical abstract

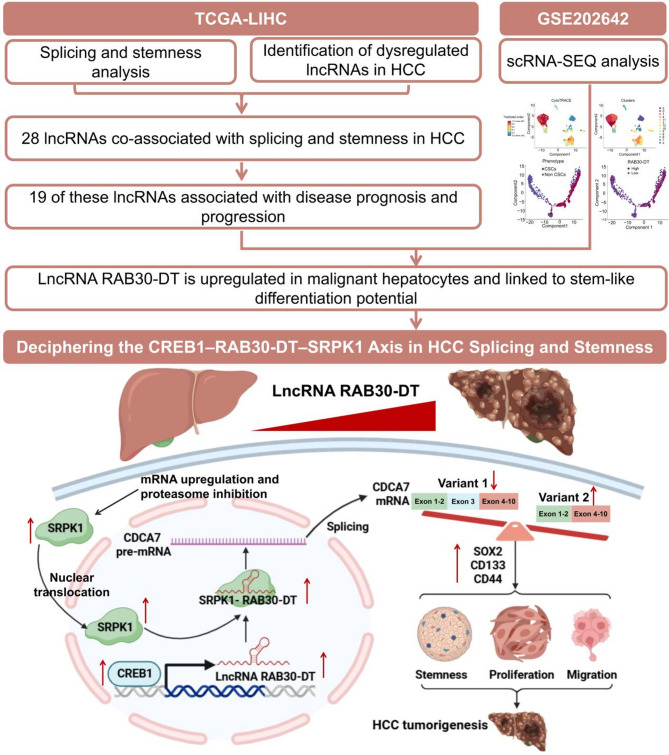

## Introduction

Hepatocellular carcinoma (HCC) is an aggressive malignancy with increasing incidence and mortality, posing a major threat to global public health [1]. Although surgical resection remains an effective treatment, the insidious onset of HCC results in most patients being diagnosed at advanced stages. Consequently, the postoperative recurrence rate reaches 70–80%, posing significant challenges for early diagnosis and effective intervention [2, 3].

Cancer stem cells (CSCs), a subpopulation within tumors with self-renewal and pluripotency capabilities, have emerged as crucial drivers of HCC recurrence, metastasis, and therapeutic resistance [4–6]. Their persistence following therapy often leads to relapse and resistance to conventional treatments, making them pivotal targets for improving therapeutic efficacy [4–6]. However, the molecular mechanisms sustaining CSC properties and tumor stemness in HCC remain largely elusive. A deeper understanding of these mechanisms is urgently needed to inform the development of effective strategies to eradicate CSCs and prevent disease relapse.

Aberrant alternative splicing (AS) represents a fundamental mechanism of transcriptome diversity and is increasingly recognized as a hallmark of cancer [7, 8]. Orchestrated primarily by splicing factors and splicing-related kinases, dysregulated AS contributes to nearly all aspects of tumor biology, including proliferation, apoptosis evasion, metabolic reprogramming, and metastasis [7, 8]. Intriguingly, recent studies suggest that AS also plays a pivotal role in maintaining CSC properties, yet the mechanisms linking AS dysregulation to tumor stemness remain poorly defined, particularly in HCC.

Long non-coding RNAs (lncRNAs), defined as transcripts exceeding 200 nucleotides without protein-coding capacity, have emerged as critical regulators of cancer development and progression [9]. In HCC, lncRNAs are increasingly implicated in promoting tumorigenesis, metastasis, and drug resistance [9, 10]. Importantly, lncRNAs exhibit multifaceted interactions with the splicing machinery: they can be AS products themselves, undergo self-splicing to produce functional isoforms, or modulate splicing by forming RNA-DNA/RNA-RNA duplexes or by altering chromatin architecture [11, 12]. Through these diverse mechanisms, lncRNAs impact key cancer-related pathways, thereby driving malignant traits such as invasion and abnormal survival [11, 12]. Despite these insights, several critical challenges remain unresolved in the field. First, there is a lack of systematic approaches to identify and characterize AS events that are functionally relevant to tumor stemness. Second, the regulatory network—particularly the contribution of lncRNAs to splicing control in CSCs—remains poorly delineated. Third, few studies have addressed how lncRNA-mediated splicing events contribute to therapy resistance and clinical outcomes. These knowledge gaps underscore the urgent need to investigate the intersection between lncRNAs, alternative splicing regulation, and tumor stemness in HCC.

In this study, we systematically investigated the interplay between AS dysregulation and stem-like phenotypes in HCC, with a specific focus on lncRNAs as potential modulators of the splicing machinery. Through integrative multi-omics analysis, we identified *RAB30-DT* as a previously uncharacterized lncRNA enriched in malignant epithelial cells with high stemness scores and poor prognosis. Although its expression has been linked to prognosis in glioblastoma [13], emerging evidence suggests a broader oncogenic role. Computational predictions further suggest that *RAB30-DT* may function as a competing endogenous RNA, sponging miR-19b-3p [14]. However, its expression dynamics, upstream regulation, and functional relevance in HCC remain unexplored. In particular, whether *RAB30-DT* participates in AS regulation and contributes to CSC-like phenotypes has not yet been investigated. Our mechanistic investigations further revealed that *RAB30-DT* directly interacts with and stabilizes serine–arginine protein kinase 1 (SRPK1), promoting its nuclear localization and driving widespread AS reprogramming, including splicing of CDCA7, a key regulator of the cell cycle and self-renewal. Furthermore, we demonstrated that *RAB30-DT* is transcriptionally activated by CREB1, establishing an lncRNA-centered regulatory axis that connects oncogenic transcriptional signaling to post-transcriptional splicing control. Importantly, pharmacological disruption of the CREB1–RAB30-DT–SRPK1 axis sensitized HCC cells to selective therapeutic compounds, highlighting its potential as a targetable vulnerability in stemness-driven HCC. These findings uncover a novel oncogenic signaling cascade that integrates lncRNA function, splicing regulation, and cancer stemness, providing mechanistic insight and therapeutic opportunities in HCC.

## Materials and methods

### Integrative analysis of splicing, stemness, and clinical associations of LncRNAs in HCC

The TCGA–LIHC dataset was obtained from TCGA database (https://portal.gdc.cancer.gov/) [15] and includes gene expression data from 374 HCC tissues and 50 adjacent normal tissues. To investigate the regulatory role of lncRNAs linking AS and cancer stemness in HCC, we first curated 167 human splicing regulatory factors from the IARA database [16] and calculated a global splicing score for each TCGA-LIHC sample as the average normalized expression (log_2_(TPM + 1.01)) of these factors. Differential expression analysis between tumor and adjacent normal tissues was conducted using the limma package (v3.56.2) in R, with thresholds of |log₂FC| >0.6 and adjusted p-value < 0.001 to identify significantly dysregulated lncRNAs. The mean expression value of the gene served as the cutoff to stratify tumor patients into high and low expression groups.

Stemness was also quantified using the mRNA stemness index (mRNAsi) algorithm, whereby the mRNA stemness index for each HCC sample was calculated based on gene expression data and subsequently normalized to a 0–1 scale using a linear transformation, following previously published methodologies [17, 18]. The mean mRNAsi value was then utilized to differentiate between high and low mRNAsi score groups. Additionally, pearson correlation analysis was used to evaluate the association between lncRNA expression and both stemness and splicing scores. For stemness, lncRNAs with correlation coefficient > 0.25 and *p* < 0.05 were considered positively associated, and those with coefficient < − 0.25 and *p* < 0.05 were considered negatively associated. For splicing score correlations, lncRNAs with correlation coefficient > 0.45 and *p* < 0.05 were defined as positively associated, and those with coefficient < − 0.25 and *p* < 0.05 as negatively associated.

The R package survival (v3.5-8) was used to perform Kaplan-Meier survival analysis with log-rank tests and two-stage procedure to assess the prognostic significance of candidate lncRNAs. A two–stage statistical approach was employed to assess significance in survival analysis. Associations with clinical characteristics—including tumor stage, metastasis status, age, gender, and ethnicity—were evaluated using the Wilcoxon rank-sum test for binary variables and the Kruskal–Wallis test for multi-category variables. Receiver Operating Characteristic (ROC) analysis, and survival analysis were conducted using the pROC (v1.18.4) [19]. This integrative approach identified lncRNAs closely linked to both molecular dysregulation and clinical outcomes in HCC, underscoring their potential roles in disease progression and prognosis.

### Genomics variation analysis

The single nucleotide variation (SNV) data for HCC were obtained from the TCGA database. After standard preprocessing and analysis, the R package ComplexHeatmap (v2.16.0) was used to generate a waterfall plot illustrating the landscape of somatic mutations. In addition, the tumor mutation burden (TMB) for each sample was calculated, and its distribution was visualized using a box plot created with the maftools package (v2.22.0).

### Analysis of scRNA-SEQ data and cell differentiation potential

A public scRNA–SEQ dataset for HCC (GSE202642) [20] was obtained from the NCBI GEO database. The data analysis and visualization were performed using the R package Seurat (version 4.03). Quality control was first performed using the following filtering criteria: 500 < nFeature_RNA < 5000, 2000 < nCount_RNA < 40,000, and percent.mt < 20. For integrated analysis across samples from different patients, the Harmony algorithm [21] was applied for batch correction. Subsequently, 20 principal components were selected for UMAP-based nonlinear dimensionality reduction [22]. Clustering was conducted with a resolution parameter set to 0.8. A low-quality cluster (Cluster 18), which co-expressed marker genes from two distinct cell types, was excluded prior to cell type annotation and downstream analyses. For analyses focused on tumor cells, dimensionality reduction and clustering were performed based on PCA results. Cells were then annotated using classical marker genes.

CytoTRACE [23] is a computational tool designed to assess the differentiation status of cells based on single-cell transcriptomic data. We further used CytoTRACE to infer the differentiation potential—or stemness level—of individual tumor cells by analyzing gene features. Tumor cells were then ordered along a differentiation trajectory. Tumor cell clusters with a CytoTRACE score greater than 0.8 were classified as CSCs, while those with scores below 0.6 were defined as Non-CSCs. Pseudotime analysis infers the developmental trajectory of cells based on dynamic changes in gene expression across different subsets. In this study, the R package Monocle (v2.28.0) [24] was used to reconstruct the differentiation trajectory of tumor cells within tumor tissues, enabling the visualization of sequential transitions and evolutionary processes among different tumor cell groups. The analysis generated corresponding pseudotime plots, dendrograms, density maps, and heatmaps. For single–cell copy number variation (CNV) analysis, the infercnv R package (v1.16.0) as used, selecting endothelial cells as the reference normal cell type. The parameters for this analysis were set as follows: cutoff = 0.1, denoise = TRUE.

### Bulk RNA–Seq data analysis and alternative splicing analysis

Total RNA was extracted using the Eastep^®^ Super Total RNA Extraction Kit (Promega, LS1040) and transported under cold chain to Beijing Novogene Technology Co., Ltd., for paired–end sequencing on the Illumina NovaSeq 6000 platform. Sequencing quality control was performed with fastp (v0.23.2) [25] to remove adapters and low–quality reads. The cleaned reads were aligned to the human reference genome (GENCODE Release 45) using STAR (v2.7.10b) [26], and the resulting alignments were sorted with samtools (v0.1.9) [27]. Gene expression was quantified using RSEM (v1.3.0) [28] and converted into a TPM matrix for further analysis.

Moreover, differential expression, Gene Ontology functional enrichment analyses were conducted using the R packages limma [29] and ClusterProfiler [30], respectively. For data visualization, the pheatmap package was used to create heatmaps, while the corrplot package (v0.92) was utilized for correlation analysis. The ggpubr package enabled the visualization of violin plots, box plots, and correlation analyses through functions such as ggviolin, ggboxplot, ggpaired, and ggscatter.

Furthermore, differential AS events were analyzed using rMATS (v4.3.0) [31], focusing on SE, MXE, RI, A5SS, and A3SS. AS events were filtered by (1) retaining those with read counts ≥ 10 in both groups, (2) excluding events with PSI values < 0.05 or > 0.95 to eliminate non–informative events, (3) ensuring FDR ≤ 0.01 to ensure statistical significance, (4) selecting events with |ΔPSI| ≥ 0.05, and (5) prioritizing genes with TPM ≥ 1. Filtered events were then analyzed via PCA, heatmaps, and functional enrichment to explore splicing regulation and its biological implications.

### Cell culture and stable cell line construction

The cell lines HepG2, Huh7, and SK–Hep–1 were obtained from the Institute of Biochemistry and Cell Biology, Shanghai Academy of Biological Sciences. These cells were cultured in DMEM (Meilunbio, China) supplemented with 10% fetal bovine serum (Meilunbio, China), 100 U/ml penicillin, and 100 µg/ml streptomycin (Beyotime, China). Culturing was performed at 37 °C in a humidified incubator with 5% CO_2_. To establish stable knockdown and overexpression cell lines, lentiviral vectors were constructed using the pLKO.1 plasmid (IGE, China). The constructs included two vectors for *RAB30-DT* knockdown (RAB30-DT–sh1 and RAB30-DT–sh2), one for *SRPK1* knockdown (SRPK1–sh), and one for *RAB30-DT* overexpression (RAB30-DT–OE). These vectors, as well as control vectors, were packaged into lentiviruses using HEK293T cells.

The resulting lentiviruses were then transduced into HepG2, Huh7, and SK–Hep–1 cell lines, followed by puromycin selection to generate stable knockdown and overexpression *RAB30-DT* cell lines. In HepG2 cell with stable *RAB30-DT* overexpression, an additional transduction with the *SRPK1* knockdown lentivirus was performed to create a cell line with simultaneous *RAB30-DT* overexpression and SRPK1 knockdown (RAB30-DT–OE–SRPK1–sh). The efficiency of *RAB30-DT* and *SRPK1* knockdown or overexpression was confirmed using quantitative PCR (qPCR), with the primer sequences provided in Table S1.

### Cell viability assay

The stable HepG2, Huh7, and SK–Hep–1 cells were separately seeded in 96–well plates at a density of 1 × 10⁴ cells per well in 180 µL of culture medium. Each experimental group included five replicates. At specified time points, 20 µL of CCK–8 reagent (Yeasen Biotechnology, China) was added to each well, and the plates were incubated for 2 h (h). Following the incubation, absorbance values were measured at 450 nm using a microplate spectrophotometer.

### Wound healing assay

The stable HepG2, Huh7, and SK–Hep–1 cells were separately seeded in 6–well plates and allowed to grow until they reached 100% confluence. A sterile 200 µL sterile gun tip was then used to create scratches in the monolayer of cells, ensuring that the scratches were perpendicular to the center of the well. After wounding, the cells were maintained in the incubator to allow for recovery and migration. Images were captured at 0 h, 12 h, and 24 h using an inverted microscope (Nikon, Japan). The average distance between the cells and the area of the wound were quantified using ImageJ software.

### Cell migration and invasion assay

Suspensions of stable HepG2, Huh7, and SK–Hep–1 cells (1 × 10^5^ cells) were added in the upper chamber of a 24–well plate (8 μm pore size, Nunc, USA) for migration assays or added to the upper chamber coated with matrix gel for invasion assays. The lower chamber was filled with 600 µL of complete medium containing 10% FBS. After 24 h of incubation at 37 °C, the upper surface of the membrane was swabbed with a cotton swab to remove remaining cells. The invaded cells in the lower chamber were fixed with 4% paraformaldehyde, stained with crystal violet (Biyuntian, China), and counted under a microscope. Four random views were selected for cell counting.

### Colony formation assay

The stable HepG2, Huh7, and SK–Hep–1 cells were cultured in 6–well culture plates (1000 cells per well) and incubated for 2 weeks until most individual cells grew into clones with > 50 cells. The cells were then fixed with methanol and stained with crystal violet solution. Colony–forming ability was assessed by counting the number of colonies (containing more than 70 cells) under a microscope. Experiments were conducted in triplicate.

### Spheroid formation assay

Cells were seeded in ultra–low attachment six–well plates at a density of 1,000 cells per well and cultured in serum–free DMEM supplemented with 2% B27 (HB319A, HUAYUN, China), 5 µg/ml insulin (40112ES25, YEASEN, China), 20 ng/ml EGF (92708ES60, YEASEN, China), and 20 ng/ml bFGF (91330ES10, YEASEN, China) for 7 to 10 days to facilitate spheroid formation. At the end of the culture period, the number of cell spheres with a diameter greater than 75 μm in each well was counted.

### RNA pull–down and mass spectrometry analysis

To prepare the DNA template for in vitro transcribed RNA, the *RAB30-DT* was cloned into the pcDNA3.1 vector with the incorporation of T7 promoters at both ends of the cloning site. Different fragments of *RAB30-DT* were amplified via PCR using primers containing the F2 fragment, and the PCR products were recovered for transcription using the T7 Quick High Yield RNA Transcription Kit (R7016S, Beyotime, China). The purity and size of the transcribed RNA were confirmed by agarose gel electrophoresis. RNA pull–down assays were conducted using the F2–RNA pull–down kit (FI8701, Fitgene, China). The proteins collected from the RNA pull–down were separated on SDS–PAGE gels, silver–stained using the Fast Silver Stain Kit (P0017S, Biotime, China), and sent to Novogene for Mass Spectrometry (MS) analysis. Western blotting was performed to validate the proteins detected by mass spectrometry.

### Western blot

Equal amounts of protein from each group were separated on SDS–PAGE and transferred to a polyvinylidene fluoride membrane. The membrane was blocked with 5% non–fat milk at room temperature for 2 h and incubated overnight at 4 °C with primary antibodies. Afterward, corresponding secondary antibodies were incubated at room temperature for 1 h. Bands were visualized using a biochemical imaging system (Amersham Imager). The antibodies used in the experiments included SRPK1 (1:1000, ProteinTech, 14073–1–AP), GAPDH (1:100,000, ProteinTech, 60004–1–Ig), HRP–conjugated Goat anti–Rabbit IgG (1:10,000, ABclonal, AS014), and HRP–conjugated Goat anti–Mouse IgG (1:10,000, ABclonal, AS003).

### Fluorescence in situ hybridization and immunofluorescence

Fluorescence in situ hybridization was performed using a kit (RiboBio, C10910). Cells on coverslips were fixed with 4% paraformaldehyde for 15 min, followed by permeabilization with PBS containing 0.2% Triton X–100 for 20 min. After the pre–hybridization solution was added, the samples were incubated at 37 °C for 30 min. The hybridization solution containing the specific probe for lncRNA RAB30-DT was added, and samples were incubated overnight at 37 °C. Following hybridization, the cells were washed six times for 5 min each with pre–warmed washing buffer.

Subsequently, immunofluorescence was performed. The cells were then blocked with 1% BSA at room temperature for 1 h and incubated overnight at 4 °C with the primary antibody against SRPK1 (1:500, ProteinTech, 14073–1–AP). After washing three times with 1 x PBS, cells were incubated with the corresponding fluorescent secondary antibody (1:1000, ProteinTech, RGAR002) at room temperature for 1–2 h. Finally, nuclei were stained with DAPI (Beyotime, C1006) for 3–5 min. Images were captured using a laser scanning confocal microscope (ZEISS 980).

### qPCR

Total RNA was extracted from HepG2, Huh7, and SK–Hep–1 cells using RNAiso Plus (Takara, Japan). According to the manufacturer’s instructions, PrimeScript™RT Kit (Takara, Japan) was utilized to reverse transcribe RNA into cDNA. For quantitative PCR (qPCR), the CFX96 Real–Time PCR system (Bio–Rad, USA) and TB green^®^Premix Ex Taq™II (Takara, Japan) were employed. The qPCR primers were listed in Table S1.

### Interaction analysis of RAB30-DT truncations with SRPK1

We used RNAComposer (https://rnacomposer.cs.put.poznan.pl/) [32, 33] to predict the tertiary structures of four truncated variants of the RAB30-DT: Δ1 (nucleotides 1–224), Δ2 (225–448), Δ3 (449–673), and Δ4 (1–448). The tertiary structure of the SRPK1 protein was retrieved from the RCSB PDB database [34] (PDB ID: pdb_00001wbp). RNA–protein interaction modeling was subsequently performed. The interaction results were visualized using PyMOL version 3.1.3.

### Xenograft assay

HepG2 cell (5 × 10^6 cells) with either knockdown of *RAB30-DT* (HepG2–RAB30-DT–sh1), overexpression of *RAB30-DT* (HepG2–RAB30-DT–OE), or control HepG2 cells were subcutaneously injected into the abdomen of 6–week–old female BALB/c nude mice (*n* = 5). Tumor size and mouse body weight were monitored throughout the study. After three weeks, the mice were euthanized, and tumors were harvested and weighed. Additionally, another set of experiments involved injecting 5 × 10^6 cells of HepG2–RAB30-DT–OE, HepG2–RAB30-DT–OE with *SRPK1* knockdown (HepG2–RAB30-DT–OE + shSRPK1) into the same mouse model (*n* = 5). Similar monitoring and harvesting procedures were followed. All animal experiments were conducted under specific pathogen–free conditions, approved by the Ethics Committee of Guangzhou Medical University (No. GY2024-387), in accordance with legal regulations and national guidelines for the care and use of laboratory animals.

### Chromatin immunoprecipitation (ChIP) assay followed by RT-PCR and qPCR

To preserve protein–DNA interactions, HepG2 cells were fixed with 1% formaldehyde for 10 min at room temperature, then quenched with 125 mM glycine. Chromatin was sheared by sonication into 200–1000 bp fragments after cell lysis. Immunoprecipitation was performed overnight at 4 °C using a CREB1-specific antibody (ProteinTech, Cat No.67927-1-Ig) or IgG control (ProteinTech, Cat No.B900620). Protein–DNA complexes were captured with Protein A/G magnetic beads and eluted after crosslink reversal. Enriched DNA containing the predicted CREB1 binding site (− 144 to − 137 bp) in the *RAB30-DT* promoter was analyzed by RT-PCR and qPCR. PCR products were confirmed by agarose gel electrophoresis, and qPCR was performed using TB Green^®^ Premix Ex Taq™ II (Takara) on a CFX96 Real-Time PCR system (Bio–Rad). Data were normalized to input and IgG controls. Primer sequences are provided in Table S1.

### Luciferase reporter assay

A 2 kb upstream region of the *RAB30-DT* promoter was synthesized (TsingKe) and cloned into the pGL4.23-basic luciferase vector (Promega). Truncations were generated by gene synthesis (TsingKe). 293T cells were co-transfected with wild-type or mutant reporter constructs and CREB1-overexpression or control plasmids using Lipofectamine 3000 (Invitrogen), along with Renilla luciferase plasmid as an internal control. Luciferase activity was measured 48 h post-transfection using the luciferase reporter gene assay kit (YEASEN,11401ES), and firefly signals were normalized to Renilla.

### Drug sensitivity analysis

As previously described [35], OncoPredict [36] was utilized to predict the drug responses of HCC patients in TCGA–LIHC based on their gene expression profiles. We conducted drug sensitivity analysis using data from the Cancer Therapeutics Response Portal 2 (CTRP2, https://portals.broadinstitute.org/ctrp.v2.1/) [37]. To experimentally validate the computational predictions, we purchased Dasatinib (HY-10181), Selumetinib (HY-50706), Daporinad (HY-50876), and Belinostat (HY-10225) from MedChemExpress (China). These compounds were used at final concentrations of 3 µM, 0.5 µM, 0.8 µM, and 0.6 µM, respectively, to treat HepG2 cells at indicated time points, following protocols reported in previous studies [38–41].

### Statistical analysis

Graphs were generated using R (v 4.3.1) or GraphPad Prism 8.0. Statistical analyses for cell viability, wound healing, cell migration and invasion, colony formation, spheroid formation, xenograft studies, qPCR, and Western blot assays were conducted using Student’s t–test. Significance levels were defined as follows: *P < 0.05; **P < 0.01; ***P < 0.001. Continuous variable data are presented as mean ± standard error of the mean.

## Results

### LncRNAs bridge aberrant alternative splicing and cancer stemness in HCC

**Fig. 1:**
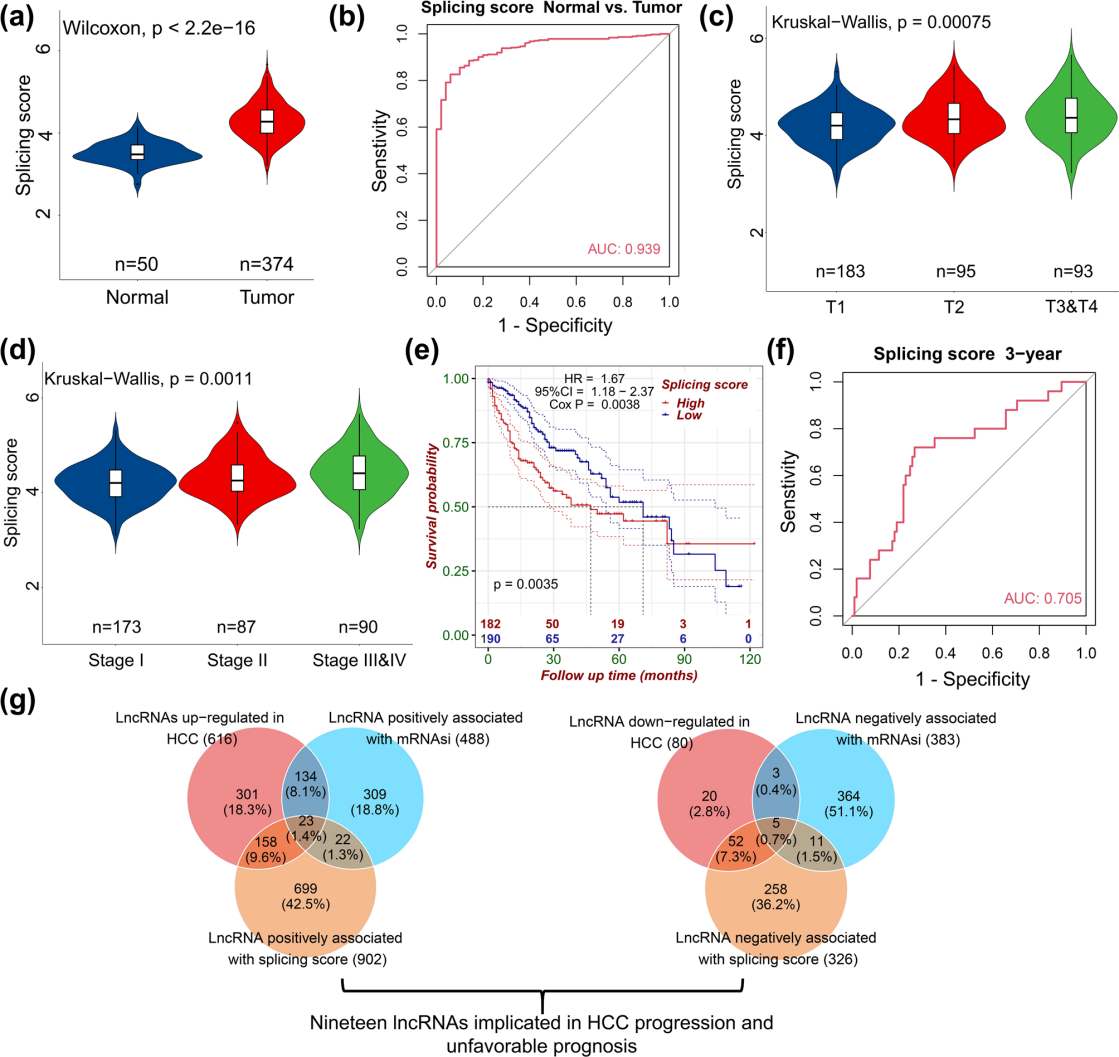
Identification of splicing- and stemness-associated lncRNAs linked to HCC progression and prognosis. **(a)** Global splicing scores, calculated as the average expression of 167 human splicing regulatory factors, are significantly elevated in HCC tumor tissues compared to adjacent normal tissues. **(b)** ROC analysis shows that splicing scores effectively distinguish tumor from normal tissues. **(c**,** d)** Splicing scores increase with HCC progression, with higher scores in late-stage tumors. **(e)** Kaplan–Meier analysis reveals that high splicing scores are associated with poorer overall survival. **(f)** Predictive value of splicing scores for 3-year survival outcomes. **(g)** Screening lncRNAs linking splicing regulation to cancer stemness in HCC

To systematically explore the role of AS in HCC development, we curated 167 human splicing regulatory factors from the IARA database [16]. Using the TCGA-LIHC dataset, we calculated a global splicing factor expression score (‘Splicing score’) for each patient, defined as the average expression level of all splicing factors in tumor tissues relative to adjacent normal tissues. Our analysis showed that splicing scores were significantly elevated in tumor tissues (Fig. 1a) and effectively distinguished tumors from normal samples as an independent factor (Fig. 1b). Notably, splicing scores increased with HCC progression, being higher in late-stage compared to early-stage tumors (Fig. 1c, d), correlated with poorer survival outcomes (Fig. 1e), and predicted 3-year survival rates (Fig. 1f**)**. These findings underscore the pivotal role of aberrant splicing regulation in HCC progression.

While dysregulated splicing has been linked to cancer stemness, the mechanisms by which lncRNAs mediate this relationship remain unclear. To address this gap, we applied the mRNA stemness index (mRNAsi) algorithm [17, 18] to quantify stemness scores in tumor and normal tissues. Through differential expression and correlation analyses, we identified 28 lncRNAs closely associated with splicing regulation, stemness, and HCC (Fig. 1g). Among these, 23 lncRNAs were upregulated and positively correlated with both splicing and stemness scores, whereas 5 were downregulated with inverse correlations (Fig. 1g). Importantly, survival analysis revealed that 19 of these lncRNAs were significantly associated with patient prognosis and disease progression (Table 1). Together, our results reveal a previously underappreciated lncRNA network that links aberrant AS to cancer stemness in HCC, providing new insights into the molecular mechanisms driving tumor progression and highlighting potential targets for therapeutic intervention.

**Table 1:** 19 LncRNAs related to splicing and stemness are significantly correlated with HCC prognosis and disease progression in the TCGA-LIHC cohort

Gene symbol	Normal vs. Tumor	Survival	T satge (T1 vs. 2 vs. 34)	Clinical satge (I vs. II vs. III&IV)	*N* stage (N0 vs. N1)	M stage (M0 vs. M1)	Age ( < = 60 vs. >60)	Gender	Race
MAPKAPK5-AS1	**** (Up)	**** (Poor)	**** (Up)	*** (Up)	-	-	-	-	-
AC004816.1	**** (Up)	*** (Poor)	**** (Up)	**** (Up)	-	-	-	-	* (Up)
SNHG3	**** (Up)	*** (Poor)	*** (Up)	*** (Up)	-	-	* (Down)	-	-
GIHCG	**** (Up)	*** (Poor)	** (Up)	** (Up)	-	-	-	-	-
AP001469.3	**** (Up)	** (Poor)	*** (Up)	*** (Up)	-	-	* (Down)	* (Down)	** (Up)
AC026401.3	**** (Up)	** (Poor)	**** (Up)	*** (Up)	-	-	-	-	-
AC145207.5	**** (Up)	** (Poor)	** (Up)	** (Up)	-	-	-	-	** (Up)
DNAJC9-AS1	**** (Up)	** (Poor)	* (Up)	* (Up)	-	-	-	-	-
ARIH2OS	**** (Up)	** (Poor)	* (Up)	-	-	-	* (Down)	* (Down)	-
AC022007.1	**** (Up)	** (Poor)	*** (Up)	** (Up)	-	-	-	-	* (Up)
SNHG20	**** (Up)	** (Poor)	*** (Up)	** (Up)	-	-	-	-	-
SNHG4	**** (Up)	** (Poor)	** (Up)	* (Up)	-	-	-	-	** (Up)
RAB30-DT	**** (Up)	** (Poor)	** (Up)	* (Up)	-	-	-	* (Down)	-
MAFG-DT	**** (Up)	* (Poor)	*** (Up)	*** (Up)	-	-	-	-	* (Up)
SCAT2	**** (Up)	* (Poor)	** (Up)	** (Up)	-	-	-	-	-
ZBTB11-AS1	**** (Up)	* (Poor)	** (Up)	** (Up)	-	-	-	-	-
AL357079.3	**** (Up)	* (Poor)	* (Up)	-	-	-	-	-	-
AC092384.2	**** (Down)	* (Better)	-	-	-	-	-	-	** (Up)
AC004160.2	**** (Down)	** (Better)	* (Down)	* (Down)	-	-	* (Up)	** (Up)	* (Up)

*: *p* ≤ 0.05; **: *p* ≤ 0.01; ***: *p* ≤ 0.001; ****: *p* ≤ 0.0001; -:No significance

### LncRNA RAB30-DT is upregulated in advanced HCC and associated with poor prognosis

**Fig. 2:**
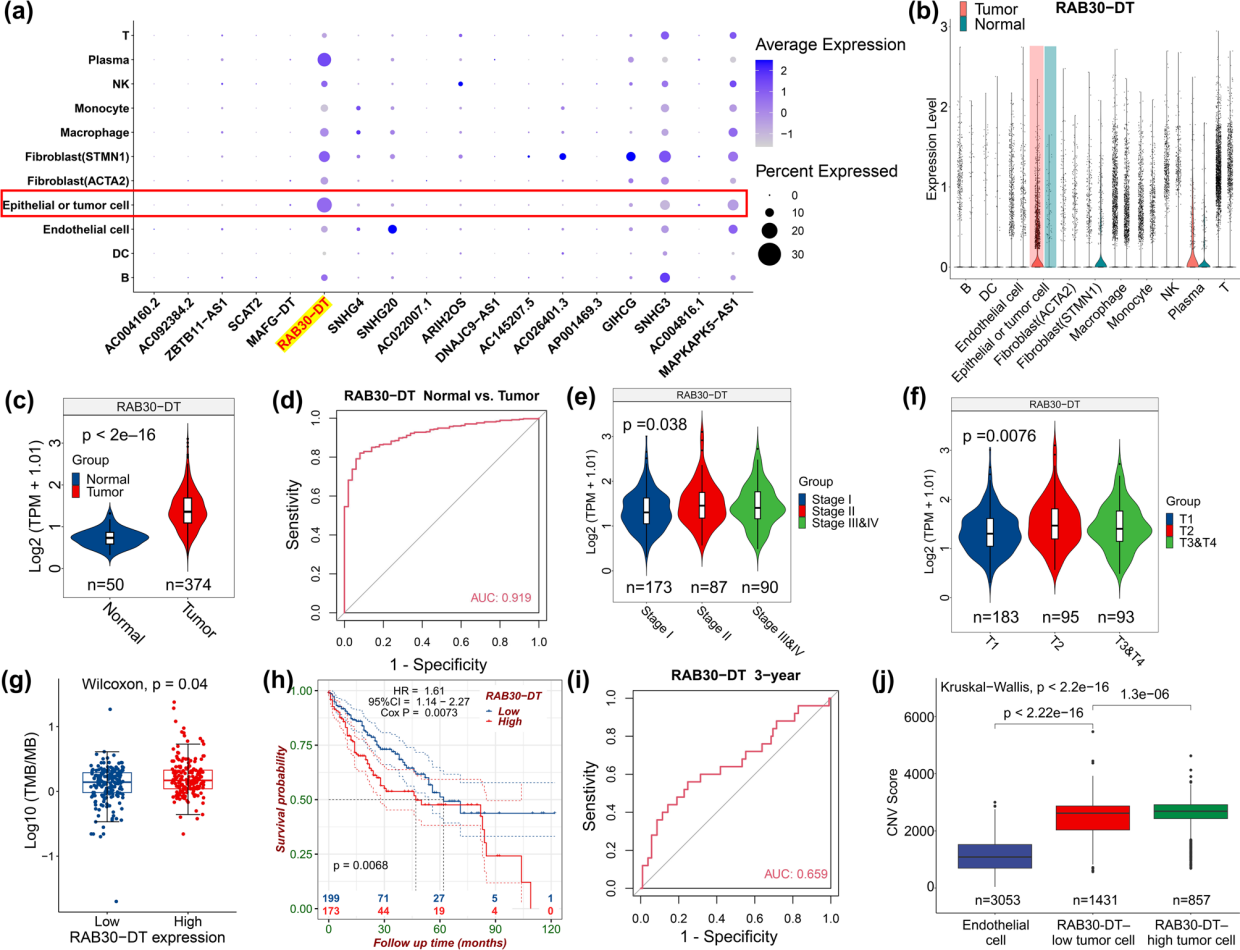
Upregulation of lncRNA RAB30-DT is associated with HCC progression and poor prognosis. **(a)** Dotplot showing Cell type-specific expression of the 19 splicing- and stemness-related lncRNAs in HCC tissues based on scRNA-SEQ analysis (GSE202642). **(b)** LncRNA *RAB30-DT* is highly expressed in tumor epithelial cells and plasma cells, but downregulated in STMN1 + fibroblasts. **(c)*** RAB30-DT* expression is significantly upregulated in HCC tissues compared to normal tissues in the TCGA-LIHC dataset. **(d)** ROC curve analysis shows the diagnostic value of *RAB30-DT* for distinguishing HCC from normal tissues. **(e**,** f)*** RAB30-DT* expression is higher in late–stage HCC (Stage III–IV and T3–T4) compared to early–stage disease. **(g)** HCC patients with high *RAB30-DT* expression exhibit higher tumor mutation burden (TMB). **(h**–**i)** High *RAB30-DT* expression is associated with greater tumor mutation burden, shorter overall survival, and serves as a predictor of 3-year survival outcomes. **(j)** Patients with high RAB30-DT expression exhibit elevated InferCNV scores in tumor epithelial cells, indicating increased genomic instability

Given the cellular heterogeneity of HCC tissues, we analyzed a single-cell RNA-SEQ (scRNA-SEQ) dataset (GSE202642) [20] to determine the cell type–specific expression patterns of the 19 splicing- and stemness-related lncRNAs in HCC (Fig. 2a and Supplementary Fig. 1a–d). The results revealed marked cell specificity for these lncRNAs. Notably, *RAB30-DT* was predominantly expressed in tumor epithelial cells, plasma cells, and STMN1-positive tumor-associated fibroblasts (Fig. 2a**)**. Similarly, *GIHCG* and *AC026401*.3 were specifically enriched in STMN1-positive fibroblasts, whereas *SNHG20* was primarily expressed in endothelial cells (Fig. 2a**)**. Further analysis showed that *RAB30-DT* expression was significantly upregulated in epithelial tumor cells and plasma cells in HCC tissues compared to adjacent normal tissues, but downregulated in STMN1-positive fibroblasts (Fig. 2b and Supplementary Fig. 1d). These findings suggest a potential role of *RAB30-DT* in HCC initiation and progression.

Consistent with the single-cell findings, analysis of the TCGA-LIHC dataset confirmed that *RAB30-DT* is significantly overexpressed in HCC tissues (Fig. 2c) and effectively distinguishes tumor from adjacent normal tissues (Fig. 2d). Its expression is markedly higher in late-stage tumors (Fig. 2e, f), and is associated with higher tumor mutation burden (TMB) (Fig. 2g), which has been linked to immunotherapy responses [42], worse prognosis (Fig. 2h), and reduced 3-year survival rates (Fig. 2i). Stratified analyses revealed that *RAB30-DT* expression correlated significantly with patient gender (Supplementary Fig. 2a), but not with age, lymph node status, distant metastasis, or race (Supplementary Fig. 2b–e). Importantly, HCC patients with high *RAB30-DT* expression frequently harbored *TP53* mutations (Supplementary Fig. 3). Supporting this, single-cell copy number variation (CNV) analysis revealed that tumor epithelial cells with elevated *RAB30-DT* expression exhibited significantly higher CNV levels (Fig. 2j), suggesting a potential role in promoting genomic instability.

Evolutionary conservation analysis using BLAST (https://blast.ncbi.nlm.nih.gov/Blast.cgi) and Clustal Omega (https://www.ebi.ac.uk/jdispatcher/msa/clustalo) further demonstrated that *RAB30-DT* is highly conserved among primates, especially in *Pan troglodytes* and *Pan paniscus*, the closest relatives to humans (Supplementary Fig. 4a, b). This conservation supports a potentially important biological function in primates. These findings highlight the splicing- and stemness-related *RAB30-DT* as a tumor-specific lncRNA associated with advanced disease, poor prognosis, TMB, *TP53* mutations, and genomic instability in HCC, suggesting it may serve as a novel biomarker and therapeutic target.

### LncRNA RAB30-DT promotes HCC development in vitro and in vivo

Studies suggest that lncRNAs can encode microproteins to regulate tumor progression [35, 43]. Notably, *RAB30-DT* has been annotated in the TransLnc database [44] as an lncRNA with potential coding capacity. To assess this potential for *RAB30-DT*, we firstly cloned and overexpressed its ORF in HCC cells, confirming that it does not encode protein (Supplementary Fig. 5a) and is localized primarily in the nucleus (Supplementary Fig. 5b). These findings suggest that *RAB30-DT* likely exerts its functions through nuclear mechanisms as an lncRNA, although further investigation is warranted.

**Fig. 3:**
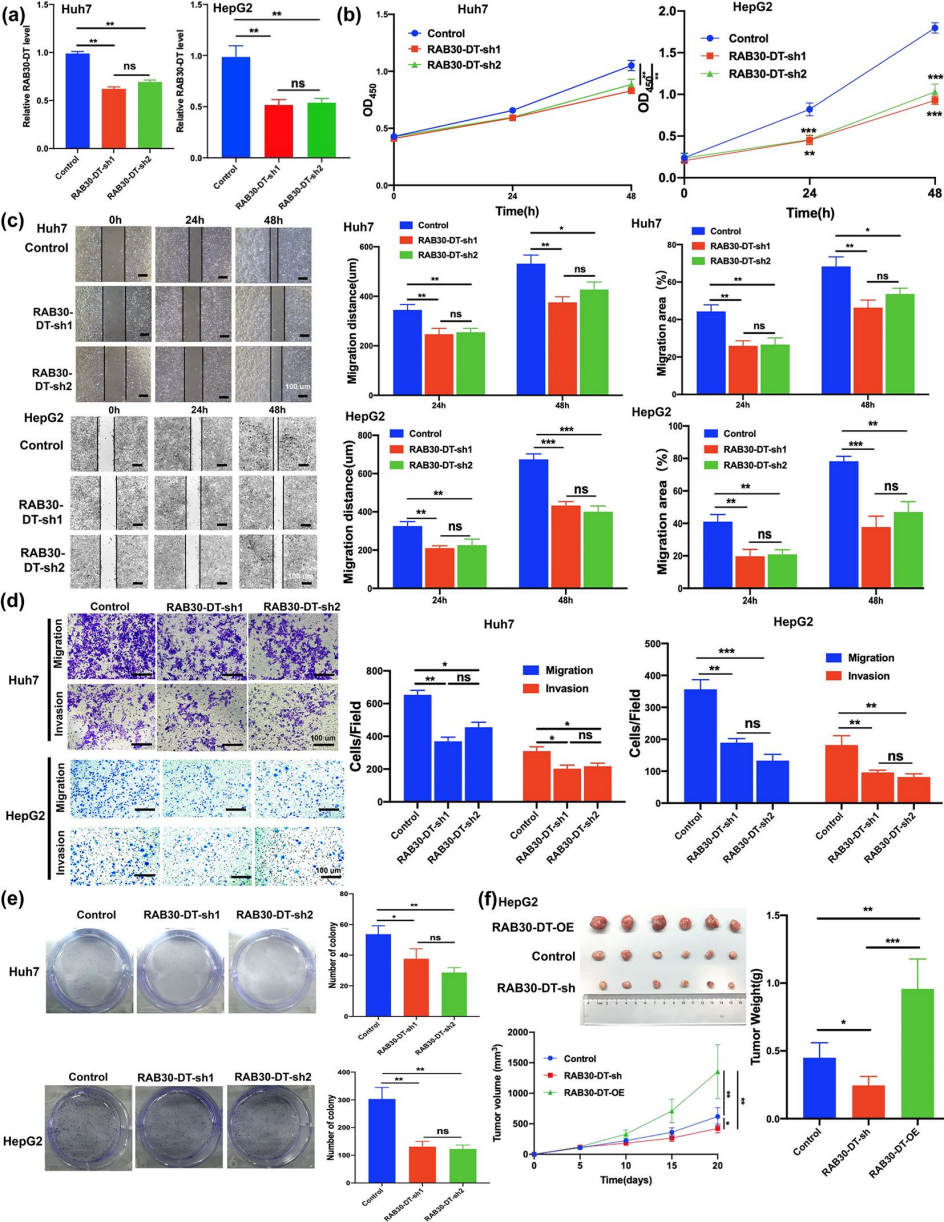
LncRNA RAB30-DT promotes HCC proliferation, migration, invasion, and tumorigenesis in vitro and in vivo. **(a)** Efficiency of *RAB30-DT* knockdown confirmed by qRT–PCR in Huh7 and HepG2 cells. **(b–d)** CCK–8 proliferation assay, wound healing assay, and Transwell assay reveal that *RAB30-DT* knockdown suppresses proliferation, migration, and invasion in Huh7 and HepG2 cells. **(e)** Colony formation assay shows reduced clonogenic capacity after *RAB30-DT* knockdown in Huh7 and HepG2 cells. **(f)** In vivo xenograft assay demonstrates that *RAB30-DT* knockdown suppresses, while overexpression promotes, tumor growth in nude mice

To experimentally assess the function of *RAB30-DT* in HCC, we constructed shRNA vectors targeting it and stably knocked down its expression in HepG2 and Huh7 cell lines (Fig. 3a). A series of cellular experiments demonstrated that *RAB30-DT* knockdown significantly inhibited HCC cell proliferation (Fig. 3b), reduced wound healing ability (Fig. 3c), and decreased migration and invasion capacities (Fig. 3d). Colony formation assays showed a marked reduction in clonogenic potential following *RAB30-DT* knockdown (Fig. 3e). Consistently, *RAB30-DT* knockdown also significantly suppressed proliferation, migration, invasion, and colony formation in the endothelial-derived SK-Hep-1 cell line (Supplementary Fig. 6a–e). In vivo, xenograft experiments in mice confirmed that *RAB30-DT* knockdown significantly reduced the tumorigenicity of HCC cells, while its overexpression enhanced (Fig. 3f and Supplementary Fig. 6f). These *in vitro and in vivo* results consistently suggested that lncRNA *RAB30-DT* functions as a potential oncogene in promoting HCC tumorigenesis.

### LncRNA RAB30-DT promotes tumor stemness in HCC

To investigate the mechanism of lncRNA *RAB30-DT* in promoting HCC tumorigenesis, we further analyzed the TCGA–LIHC dataset. The analysis results confirmed that high expression of *RAB30-DT* in HCC tissues activated stemness– and development–related pathways (Supplementary Fig. 7a–c) and correlated with higher mRNAsi scores, indicating increased tumor stemness (Supplementary Fig. 7d). Consistently, HCC tissues with high mRNAsi scores exhibited significantly elevated *RAB30-DT* expression (Supplementary Fig. 7e), with a strong positive correlation between the two (Supplementary Fig. 7f–g). Additionally, *RAB30-DT* expression in HCC was positively correlated with eight validated CSC–related genes (\Supplementary Fig. 7h), which were significantly enriched in pathways associated with stemness and cellular development (Supplementary Fig. 7a–b). These findings suggest that lncRNA *RAB30-DT* contribute to maintaining tumor stemness in HCC.

**Fig. 4:**
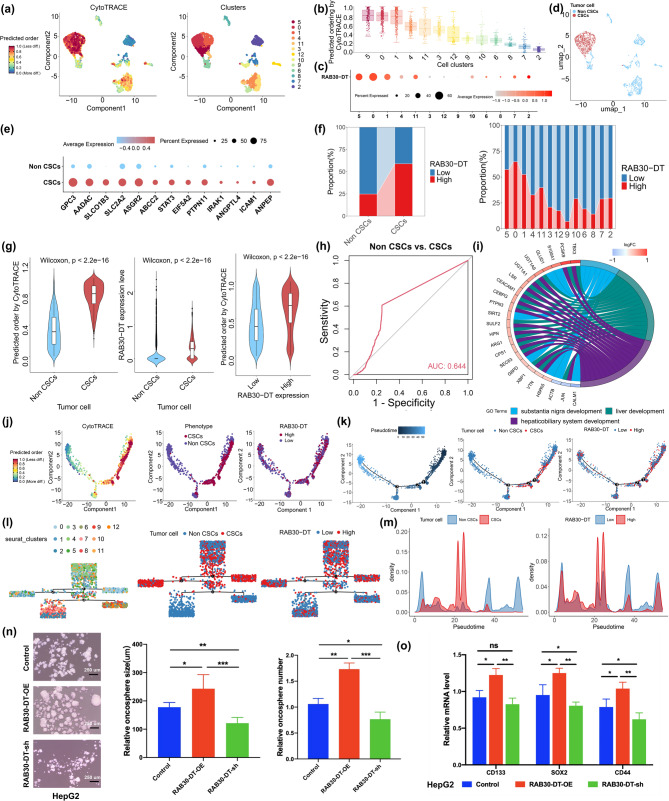
LncRNA RAB30-DT promotes tumor cell stemness in HCC. **(a)** UMAP showing CytoTRACE scores across tumor epithelial cells, indicating differentiation potential. **(b)** Bar plot of CytoTRACE scores across tumor cell clusters. **(c)** Dot plot of *RAB30-DT* expression in each cluster. **(d)** UMAP distinguishing CSC–like and non–CSC populations based on CytoTRACE scores. **(e)** Dot plot of stemness–related gene expression in CSC–like versus non–CSC populations. **(f)** Stacked bar chart showing elevated *RAB30-DT* expression in CSC–like and CytoTRACE–high cells. **(g)** Violin plots showing higher CytoTRACE scores and *RAB30-DT* expression in CSC–like cells, and a positive correlation between *RAB30-DT* expression and CytoTRACE score. **(h)** ROC analysis showing *RAB30-DT* effectively discriminates CSC–like from non–CSC cells. **(i)** GO enrichment analysis of *RAB30-DT*–high cells reveals activation of liver development pathways. **(j–m)** Pseudotime analysis linking *RAB30-DT* expression to malignant cell differentiation states. **(n)** Tumorsphere assays show that *RAB30-DT* overexpression promotes, while knockdown impairs, HepG2 sphere formation. **(o)** qPCR analysis of *CD133*, *SOX2*, and *CD44* expression in HepG2 spheroids following RAB30-DT knockdown or overexpression

Considering the cellular heterogeneity of HCC tissue, we further investigated the impact of *RAB30-DT* on tumor stemness at the single–cell level. Using the scRNA–SEQ data and the CytoTRACE [23] tool to assess differentiation potential, we annotated CSC and non–CSC cells in HCC (Fig. 4a–e). Further analysis confirmed that *RAB30-DT* was significantly upregulated in tumor cells with high differentiation potential, where it played a key role in maintaining tumor stemness (Fig. 4b–e). Further analysis revealed that *RAB30-DT* expression was higher in CSCs compared to non–CSCs, and was associated with higher differentiation potential scores, helping to distinguish CSCs from non–CSCs (Fig. 4f–h). Functional enrichment analysis indicated that high expression of *RAB30-DT* upregulated liver development–related pathways in HCC tumor cells (Fig. 4i).

In line with these findings, further pseudotime analysis of tumor cells confirmed that the expression of *RAB30-DT* strongly associated with the differentiation potential of malignant tumor cells (Fig. 4j–m and Supplementary Fig. 8). In addition, tumorsphere formation assays demonstrated that overexpression of *RAB30-DT* significantly enhanced tumorsphere-forming ability and upregulated the expression of stemness genes *CD133*, *SOX2*, and *CD44*, whereas *RAB30-DT* knockdown produced the opposite effect (Fig. 4n–o). ALDH (Aldehyde Dehydrogenase) activity is a functional marker of CSCs [45]. To investigate whether *RAB30-DT* regulates ALDH activity, we observed cell line–dependent effects. Specifically, knockdown of *RAB30-DT* significantly reduced *ALDH1A1* expression in HepG2 cells but had no appreciable effect in Huh7 cells, whereas *ALDH2* expression remained largely unchanged in both cell lines (Supplementary Fig. 9a). These results suggest that ALDH genes may not serve as universal mediators of *RAB30-DT*–driven CSC-like phenotypes, and that this process may instead be more strongly influenced by factors such as *CD133*, *SOX2*, and *CD44*. Collectively, our findings indicate that lncRNA *RAB30-DT* plays a pivotal role in promoting and maintaining tumor stemness, thereby contributing to HCC progression.

### LncRNA RAB30-DT orchestrates splicing reprogramming to drive tumor stemness and progression in HCC

**Fig. 5:**
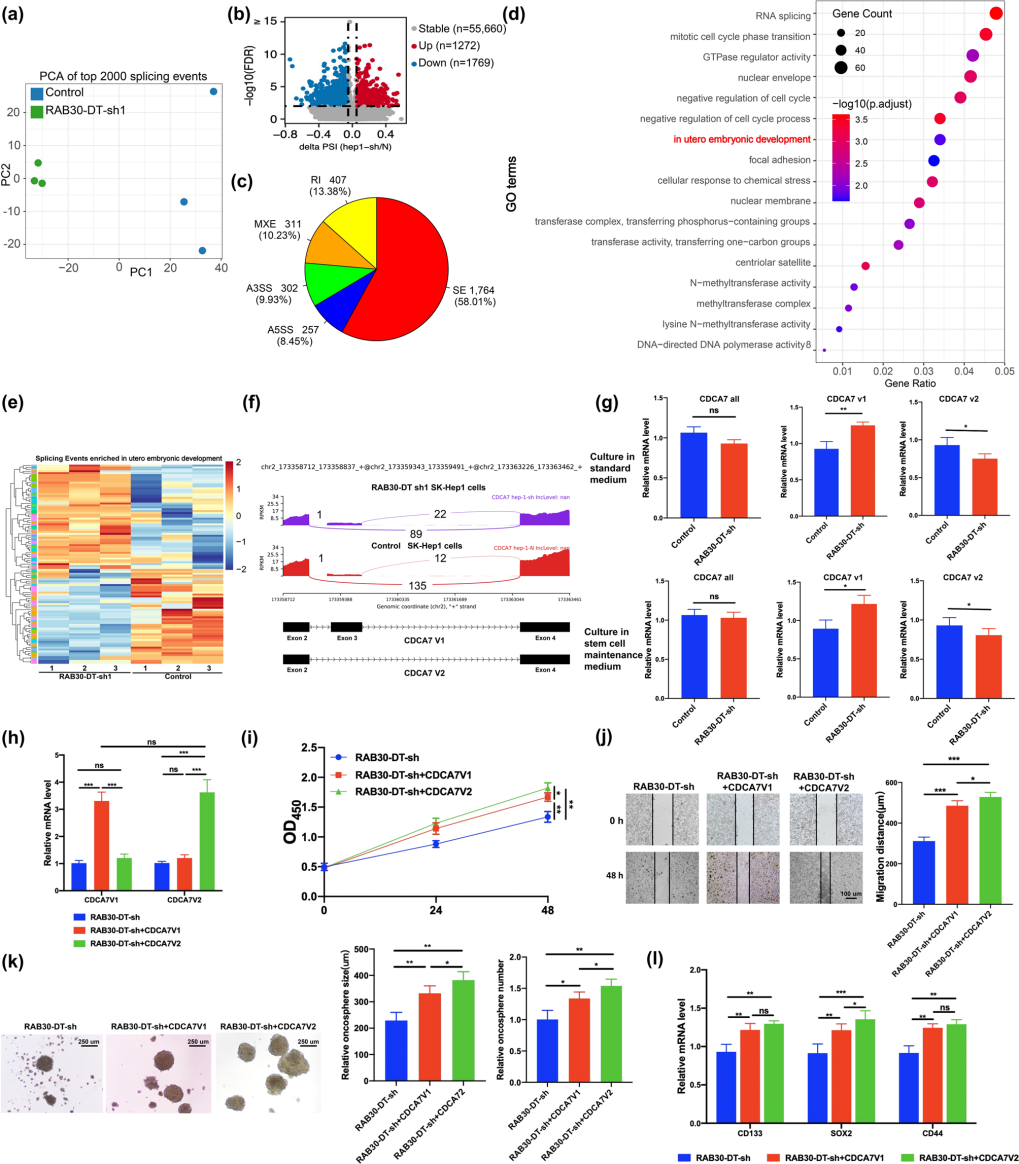
LncRNA RAB30-DT orchestrates splicing reprogramming to drive tumor stemness and progression. **(a)** principal component analysis shows distinct AS profiles in SK-Hep-1 cells upon *RAB30-DT* knockdown. **(b)** Volcano plot of significantly altered AS events upon *RAB30-DT* depletion. **(c)** Classification of differential AS events: SE, RI, MXE, A3SS, and A5SS. **(d)** GO enrichment reveals involvement in development and cell cycle pathways. **(e)** Heatmap of AS events enriched in developmental pathways. **(f**,** g)** Sashimi plot and qPCR confirm reduced *CDCA7* exon 3 skipping after *RAB30-DT* knockdown. **(h)** qPCR validation of *CDCA7* variant 1 (V1) and variant 2 (V2) overexpression in RAB30-DT–sh HepG2 cells. **(i–k)*** CDCA7* splicing variants rescue *RAB30-DT* knockdown–induced suppression of HepG2 cell proliferation, migration, and stemness. **(l)** qPCR analysis of *SOX2*, *CD133*, and *CD44* expression in response to RAB30-DT knockdown and CDCA7 V1/V2 overexpression

To elucidate the molecular mechanisms by which lncRNA *RAB30-DT* promotes tumor stemness and HCC progression, we performed RNA–Seq analysis on HCC cells following *RAB30-DT* knockdown. The results showed that silencing *RAB30-DT* significantly altered global gene expression (Supplementary Fig. 9b), particularly affecting pathways associated with liver development and AS regulation (**Supplementary Fig. 9c–d**), suggesting that *RAB30-DT* may modulate tumor stemness through AS. Although *RAB30-DT* knockdown did not markedly change the distribution of AS event types (Supplementary Fig. 9e–f), it extensively altered AS patterns of genes (Fig. 5a). Differential AS analysis identified 3,041 significantly affected AS events, including 1,272 upregulated and 1,769 downregulated (Fig. 5b). Among these, skipped exons (SE) events were most prevalent (58.01%), followed by retained introns (RI) (13.38%), mutually exclusive exons (MXE) (10.23%), alternative 3’ splice sites (A3SS) (9.93), and alternative 5’ splice sites (A5SS) (8.45%) events (Fig. 5c).

Functional enrichment analysis revealed that these differential AS events were significantly enriched in embryonic development and cell cycle pathways (Fig. 5d), with strong functional coherence among the involved AS events (Fig. 5e and Supplementary Fig. 9g). Notably, while *RAB30-DT* knockdown did not alter the overall expression of *CDCA7*, it reduced exon 3 skipping, leading to an increase in *CDCA7* variant 1 and a decrease in variant 2 (Fig. 5f–g). Consistent with our findings, *CDCA7* has been identified as a potential oncogene involved in embryonic development and the regulation of stemness [46–48]. Further experiments demonstrated that while overexpression of both *CDCA7* splice variants could alleviate the inhibitory effects of *RAB30-DT* knockdown on HCC cell proliferation, migration, and stemness, variant 2 exhibited a markedly stronger rescue effect than variant 1 (Fig. 5h–k). These results suggest that lncRNA *RAB30-DT* enhances tumor stemness and promotes HCC progression by regulating the mRNA AS of *CDCA7*.

### LncRNA RAB30-DT promotes tumor stemness and HCC progression via SRPK1–mediated CDCA7 alternative splicing

**Fig. 6:**
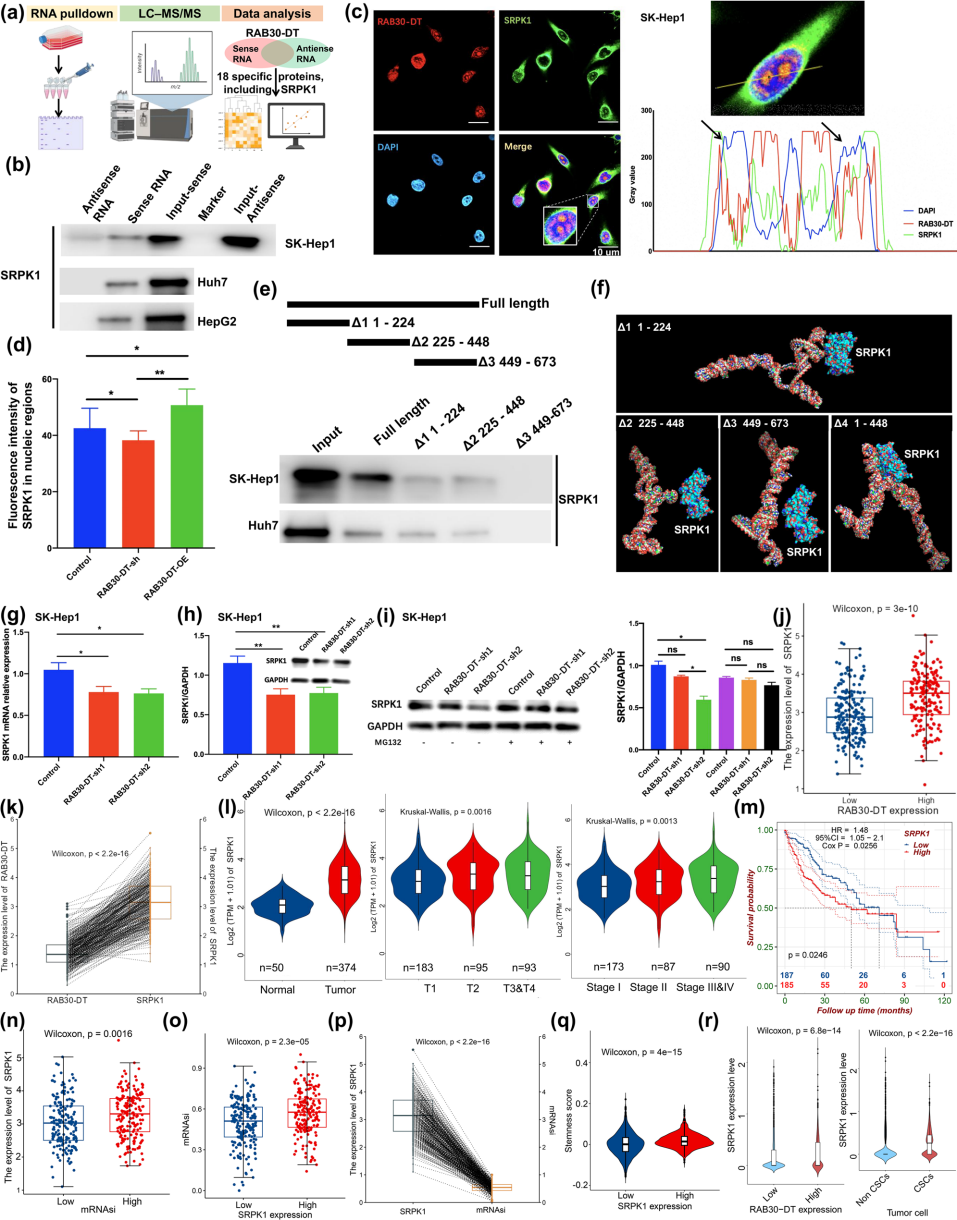
LncRNA RAB30-DT upregulates SRPK1 expression and directly interacts with its in nucleus. **(a)** RNA pull–down proteomics workflow to identify binding partners of *RAB30-DT*. **(b)** RNA pull–down assays confirm the specific interaction between *RAB30-DT* and SRPK1 in HepG2, Huh7, and SK-Hep-1 cells. **(c)** FISH and immunofluorescence confirm co–localization of *RAB30-DT* and SRPK1 in the nucleus. **(d)*** RAB30-DT* knockdown reduces nuclear SRPK1 protein levels, whereas its overexpression increases nuclear SRPK1 levels. **(e)** Truncation analysis identifies the SRPK1–binding region on *RAB30-DT* in Huh7 and SK-Hep-1 cells. **(f)** PyMol visualization shows the binding interaction between SRPK1 and various truncation mutants of *RAB30-DT.* **(g–h)*** RAB30-DT* knockdown leads to decreased SRPK1 mRNA and protein expression. **(i)*** RAB30-DT* knockdown enhances SRPK1 proteasomal degradation. **(j–k)** SRPK1 expression is significantly elevated in HCC with high *RAB30-DT* expression. **(l)** SRPK1 is significantly upregulated in high–stage HCC tissues. **(m)** High SRPK1 expression is positively correlated with poor survival prognosis in HCC patients. **(n–q)*** SRPK1* expression is positively correlated with mRNAsi scores in HCC patients. **(r)*** SRPK1* expression is elevated in tumor cells with high *RAB30-DT* levels and in CSCs

To elucidate the molecular mechanism by which lncRNA *RAB30-DT* regulates AS and promotes HCC progression, we conducted RNA pull–down assays followed by Mass Spectrometry (MS) analysis (Fig. 6a). This approach identified 18 proteins specifically interacting with *RAB30-DT*, among which SRPK1—a key splicing regulatory kinase—was notably enriched (Fig. 6a; Supplementary Fig. 10a). Analysis of the TCGA–LIHC dataset revealed a strong correlation between *RAB30-DT* and the expression of these 18 candidate genes, supporting the reliability of the pull–down results (Supplementary Fig. 10b–d). Further RNA pull–down, fluorescence in situ hybridization (FISH), and immunofluorescence assays confirmed that *RAB30-DT* directly interacts with SRPK1 in the nucleus (Fig. 6b, c). Notably, knockdown of *RAB30-DT* reduced the nuclear localization of SRPK1 (Fig. 6d). Truncation analysis mapped the RAB30-DT–SRPK1 interaction to the 1–448 nt region of *RAB30-DT* (Fig. 6e), and this interaction was further validated through simulated docking analysis (Fig. 6f). Mechanistically, *RAB30-DT* depletion not only decreased SRPK1 mRNA levels but also promoted its proteolytic degradation, indicating a dual level of regulation (Fig. 6g–i). Consistently, TCGA–LIHC data revealed a positive correlation between *SRPK1* and *RAB30-DT* expression (Fig. 6j, k). Elevated *SRPK1* levels were associated with HCC progression (Fig. 6l, m and Supplementary Fig. 11a–g), as well as with higher mRNAsi scores and increased tumor cell stemness in HCC patients (Fig. 6n–r). Moreover, pseudotime trajectory analysis revealed that *SRPK1* expression is closely linked to the differentiation potential of malignant tumor cells in HCC, further highlighting its role in tumor development (Supplementary Fig. 11h).

**Fig. 7:**
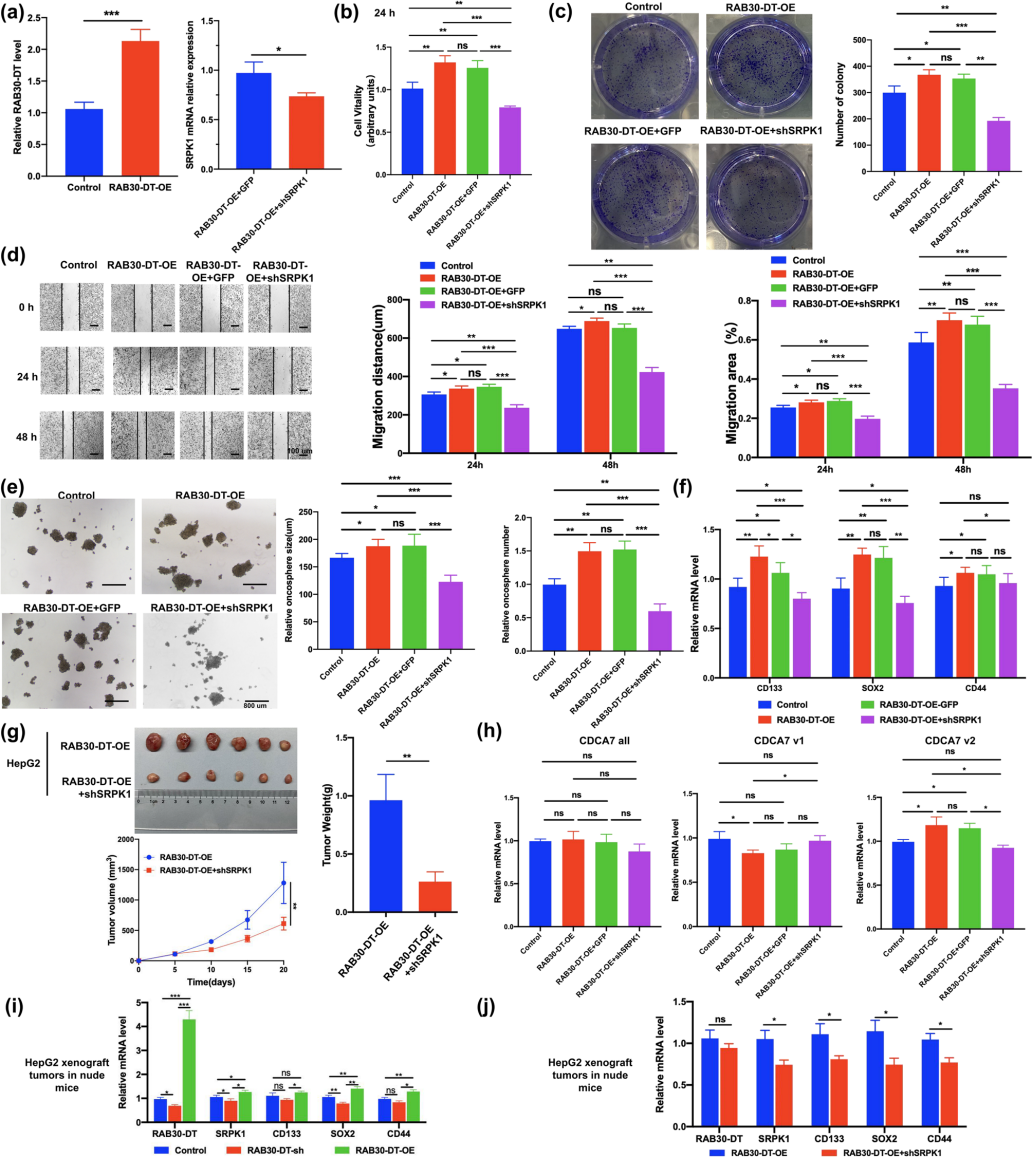
SRPK1 is essential for RAB30-DT–mediated tumor progression and stemness through regulating CDCA7 splicing and the expression of ***SOX2***, ***CD133***, and ***CD44***. **(a)** Validation of *RAB30-DT* overexpression and *SRPK1* knockdown efficiency in HepG2 cells. **(b–e)*** SRPK1* knockdown abolishes *RAB30-DT*–induced proliferation, colony formation, migration, and tumorsphere formation in HCC cells. **(f)** qPCR analysis shows that *SRPK1* knockdown suppresses *RAB30-DT*–induced upregulation of stemness markers *SOX2*, *CD133*, and *CD44*. **(g)** Xenograft assays demonstrate that *SRPK1* silencing attenuates *RAB30-DT*–induced tumor growth in vivo. **(h)** Alternative splicing analysis during tumorsphere formation shows that SRPK1 is required for *RAB30-DT*–mediated regulation of *CDCA7* splicing. **(i–j)** qPCR analysis of HepG2–derived xenograft tumors confirms that *RAB30-DT* promotes, and *SRPK1* knockdown suppresses, the expression of *SOX2*, *CD133*, and *CD44*

Rescue experiments demonstrated that the oncogenic functions of lncRNA *RAB30-DT* are dependent on *SRPK1*. Specifically, *SRPK1* knockdown reversed the *RAB30-DT*–induced enhancement of cell proliferation (Fig. 7a–b), colony formation (Fig. 7c), migration (Fig. 7d), and tumorsphere formation (Fig. 7e), as well as the upregulation of stemness–associated markers *SOX2*, *CD133*, and *CD44* (Fig. 7f). In vivo, *SRPK1* silencing also mitigated *RAB30-DT*–mediated tumor growth in nude mouse xenograft models (Fig. 7g). Mechanistically, *RAB30-DT* regulates the AS of *CDCA7* in a SRPK1–dependent manner during tumorsphere formation (Fig. 7h). Furthermore, qPCR analysis of HepG2–derived xenograft tumors confirmed that *RAB30-DT* promotes the expression of *SOX2*, *CD133*, and *CD44* through its interaction with SRPK1 (Fig. 7i, j). Collectively, these results indicate that lncRNA *RAB30-DT* enhances tumor stemness and HCC tumorigenesis by regulating *CDCA7* mRNA AS in an SRPK1-dependent manner.

### Transcription factor CREB1 mediates the upregulation of lncRNA RAB30-DT in HCC

**Fig. 8:**
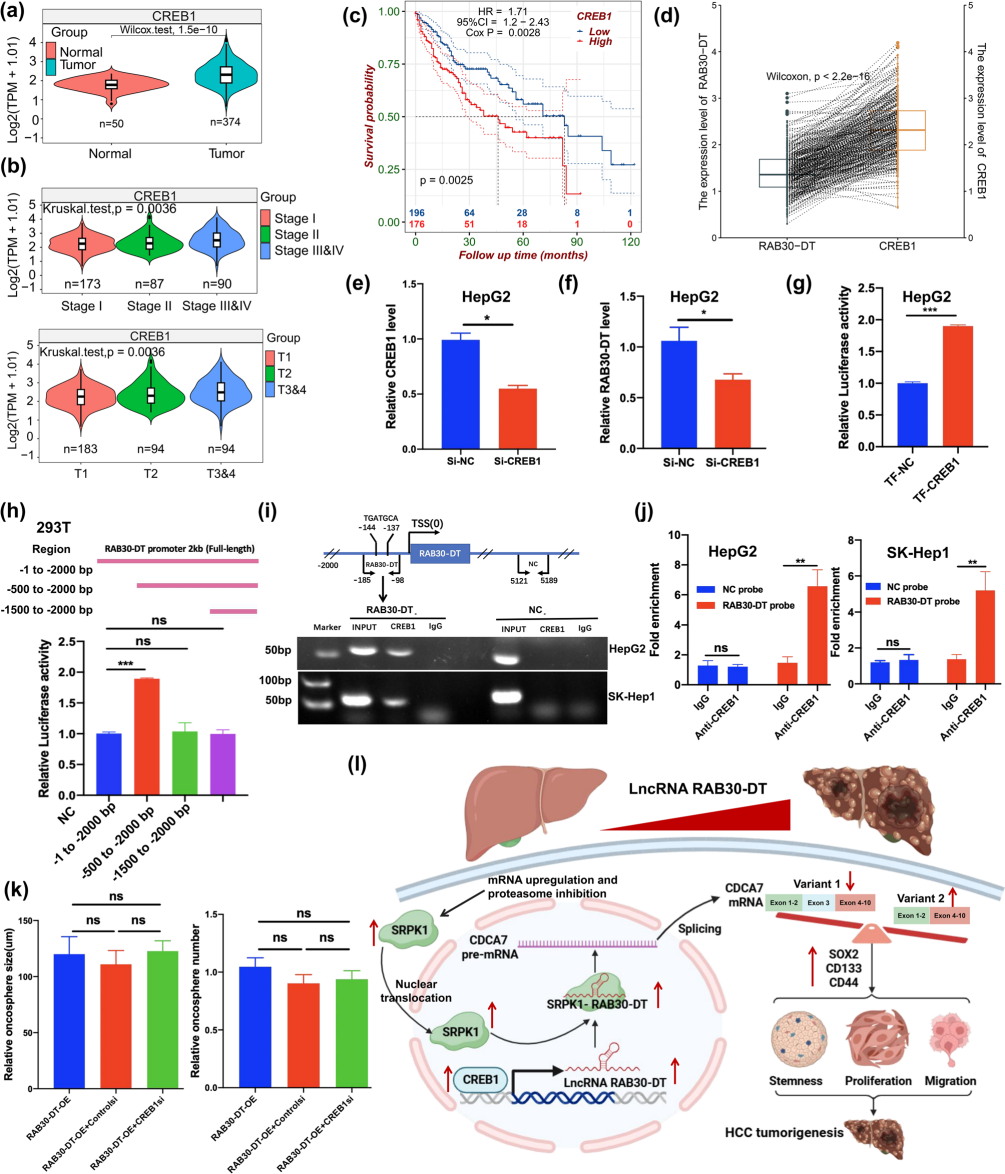
Transcription factor CREB1 drives the transcriptional upregulation of lncRNA RAB30-DT in HCC. **(a–c)** CREB1 is upregulated in HCC and is associated with disease progression and poor prognosis. **(d)** The gene expression level of *CREB1* shows a positive correlation with that of *RAB30-DT*. **(e–f)** Knockdown of *CREB1* in HepG2 cells significantly reduces *RAB30-DT* expression. **(g)** Overexpression of *CREB1* enhances luciferase activity driven by the *RAB30-DT* promoter. **(h)** Co–transfection of CREB1 and *RAB30-DT* promoter truncation constructs identifies the − 1 to − 500 bp region as the CREB1–responsive element. **(i–j)** ChIP RT–PCR and qPCR confirm direct binding of CREB1 to the predicted − 144 to − 137 bp region of the *RAB30-DT* promoter. **(k)** Knockdown of *CREB1* in *RAB30-DT*–overexpressing HepG2 cells and its effect on HCC tumor stemness. **(l)** Schematic illustration of the molecular mechanism by which CREB1–induced *RAB30-DT* upregulation promotes tumor stemness and progression in HCC

cBioPortal [49] analysis showed that while lncRNA *RAB30-DT* is consistently upregulated in HCC, genomic alterations at its locus, including copy number and structural variants, are rare (< 1% of patients) (Supplementary Fig. 12a). This discrepancy suggests that *RAB30-DT* upregulation is likely driven by alternative regulatory mechanisms beyond genetic alterations. To investigate potential transcriptional regulators, we analyzed the − 2 kb promoter region of the *RAB30-DT* using TFBS predictions from the JASPAR CORE (2022) database via the UCSC Genome Browser (hg38) [50]. The top 30 TFs, ranked by binding score, were cross–referenced with TCGA–LIHC expression data (Supplementary Table S2). Among them, CREB1 emerged as one of the strong candidates, exhibiting significant upregulation in HCC and a positive correlation with both tumor progression and poor patient survival (Fig. 8a–c and Supplementary Table S2). Additionally, *CREB1* expression was positively correlated with that of *RAB30-DT* (Fig. 8d). This is consistent with previous reports identifying CREB1 as an oncogenic factor involved in promoting tumor stemness and chemoresistance [51–53]. However, whether CREB1 promotes tumor stemness through transcriptional activation of *RAB30-DT* has not been previously reported, and our findings provide novel insights into this potential regulatory axis.

To validate this regulatory relationship, we silenced *CREB1* in HCC cells using a specific siRNA (Fig. 8e), resulting in a marked reduction in *RAB30-DT* expression (Fig. 8f). Luciferase reporter assays demonstrated that *CREB1* overexpression markedly enhanced the transcriptional activity of the *RAB30-DT* promoter (Fig. 8g). Further *RAB30-DT* promoter truncation analysis pinpointed a critical regulatory region spanning − 500 to − 1 bp that is essential for CREB1–driven activation (Fig. 8h). Notably, this region contains a predicted CREB1 binding site located between − 144 and − 137 bp (Fig. 8i). Moreover, ChIP assays using specific anti–CREB1 antibodies, followed by RT–PCR and qPCR, confirmed direct CREB1 binding to this promoter region, yielding a specific 87 bp amplicon containing the predicted site (Fig. 8j). Furthermore, knockdown of CREB1 in cells overexpressing *RAB30-DT* does not attenuate the *RAB30-DT*–induced enhancement of stemness (Fig. 8j and Supplementary Fig. 12b), proliferation (Supplementary Fig. 12c), and migration (Supplementary Fig. 12b). This suggests that *RAB30-DT f*unctions downstream of CREB1 and serve as a key effector mediating CREB1-driven malignant phenotypes in HCC. Thus, these findings demonstrate that CREB1 directly binds to and activates the *RAB30-DT* promoter, leading to its upregulation, which in turn interacts with SRPK1 to regulate *CDCA7* AS and promote tumor stemness and HCC tumorigenesis (Fig. 8l).

### Discovering therapeutic strategies to target the LncRNA CREB1–RAB30-DT–SRPK1–Stemness axis in combating HCC

**Fig. 9:**
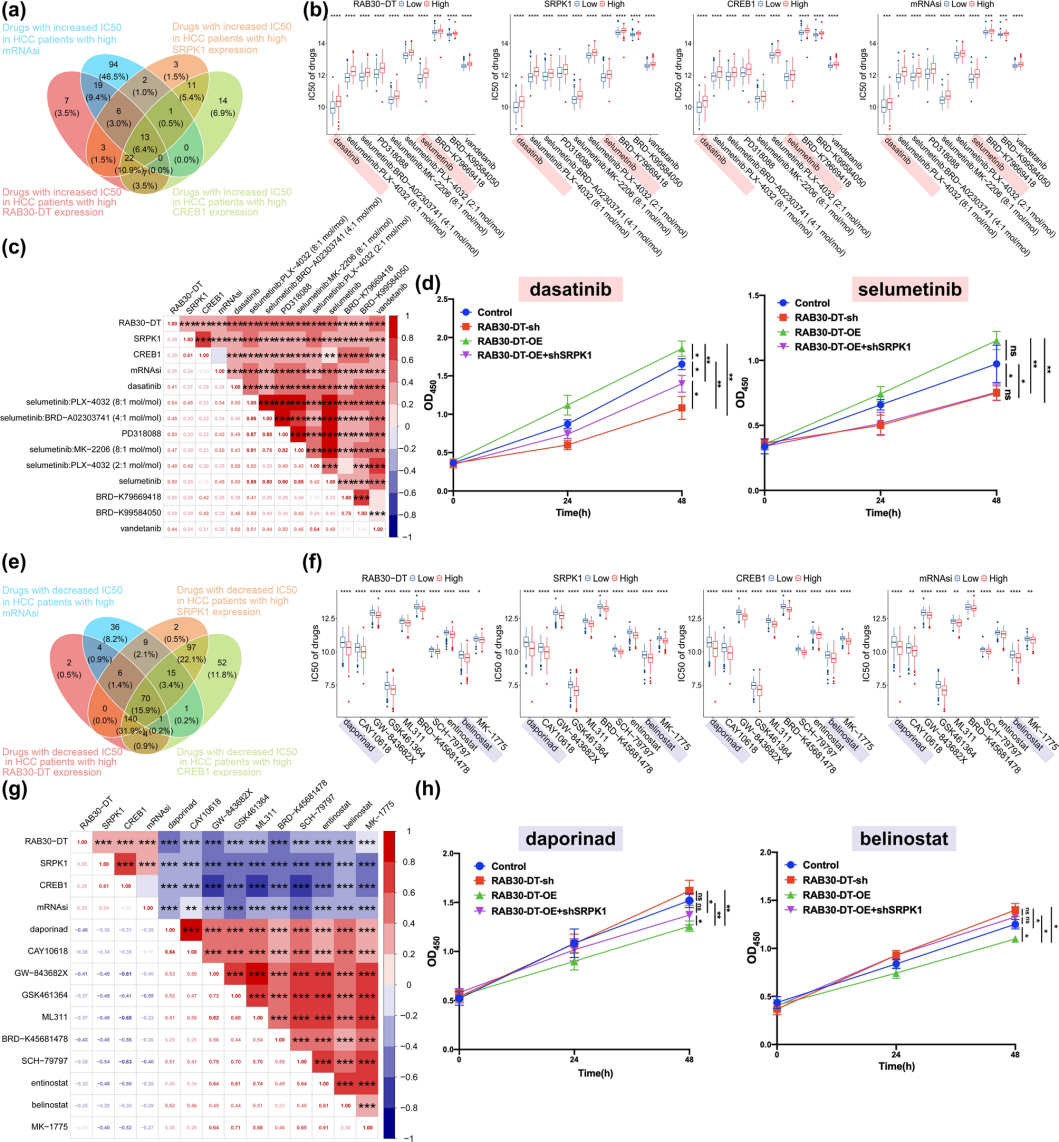
Identification of potential therapeutic agents targeting the CREB1–RAB30-DT–SRPK1–stemness axis in HCC. **(a)** Drug sensitivity analysis identified 13 compounds with significantly higher IC50 values in patients with high *RAB30-DT*, *SRPK1*, and *CREB1* expression, as well as elevated mRNAsi scores. **(b)** Top 10 drugs with the greatest increase in IC50 values in the high–expression group, suggesting reduced sensitivity. **(c)** Positive correlations between IC50 values and the expression levels of *RAB30-DT*, *SRPK1*, *CREB1*, and mRNAsi scores. **(d)** CCK–8 assays showed that *RAB30-DT* overexpression decreased the sensitivity of HepG2 cells to dasatinib (3 μm) and selumetinib (0.5 μm), while *SRPK1* knockdown reversed this reduced sensitivity. **(e)** A total of 70 compounds showed significantly lower IC50 values in the same patient group, indicating enhanced drug sensitivity. **(f)** Top 10 drugs with the greatest decrease in IC50 values in the high–expression group. **(g)** Negative correlations between IC50 values and the expression levels of *RAB30-DT*, *SRPK1*, *CREB1*, and mRNAsi scores. **(h)** CCK–8 assays demonstrated that *RAB30-DT* overexpression increased the sensitivity of HepG2 cells to daporinad (0.8 μm) and belinostat (0.6 μm), while *SRPK1* knockdown abrogated this enhanced sensitivity

To identify potential therapeutic agents targeting the CREB1–RAB30-DT–SRPK1–stemness axis, we conducted drug sensitivity analysis using the TCGA–LIHC dataset in combination with OncoPredict [36] and CTRP2 [37] tools. A total of 13 compounds were found to exhibit significantly increase in IC50 values in patients with high expression of *RAB30-DT*, *SRPK1*, and *CREB1*, as well as elevated mRNAsi scores, indicating reduced drug sensitivity in this subgroup (Fig. 9a and Supplementary Table S3). The top 10 drugs with the greatest increase in IC50 values are shown in Fig. 9b. Correlation analysis further revealed that the IC50 values of these drugs were positively associated with the expression levels of *RAB30-DT*, *SRPK1*, *CREB1*, and mRNAsi scores, suggesting limited efficacy of these agents in targeting HCC cells with strong stemness features (Fig. 9c). Consistently, CCK–8 assays demonstrated that overexpression of *RAB30-DT* reduced the sensitivity of HepG2 cells to dasatinib and selumetinib, while knockdown of *SRPK1* reversed this resistance phenotype (Fig. 9d), supporting the notion that *SRPK1* mediates RAB30-DT–induced drug insensitivity.

In contrast, 70 compounds displayed significantly decrease in IC50 values in the same high–expression patient group, suggesting enhanced drug sensitivity (Fig. 9e and Supplementary Table S3). Among them, the top 10 most potent drugs are depicted in Fig. 9f. The IC50 values of these drugs showed a negative correlation with *RAB30-DT*, *SRPK1*, and *CREB1* expression levels, as well as with mRNAsi scores, indicating that patients with enhanced activity of the CREB1–RAB30-DT–SRPK1 axis may be more responsive to these treatments (Fig. 9g). Further in vitro validation using CCK–8 assays revealed that *RAB30-DT* overexpression increased the sensitivity of HepG2 cells to daporinad and belinostat, while *SRPK1* knockdown abrogated this enhanced responsiveness (Fig. 9h), suggesting that *SRPK1* is essential for RAB30-DT–mediated modulation of drug response. Together, these findings highlight the potential of targeting the CREB1–RAB30-DT–SRPK1 axis as a therapeutic strategy to overcome tumor stemness and improve treatment efficacy in HCC.

## Discussion

HCC is among the deadliest malignancies, driven by its high heterogeneity, frequent recurrence, and limited therapeutic options at advanced stages [1, 3]. Increasing evidence implicates CSCs in promoting HCC progression, metastasis, and resistance to therapy [4, 5]. However, the molecular basis sustaining CSC-like traits remains poorly defined. Here, we uncover a previously unrecognized role of lncRNAs in linking aberrant AS with CSC-like phenotypes in HCC. Integrative analysis of bulk and single-cell transcriptomes identified 28 lncRNAs associated with both elevated stemness and splicing dysregulation, among which 19 possessed strong prognostic relevance.

Notably, lncRNA *RAB30-DT* emerged as a key oncogenic lncRNA, upregulated in malignant epithelial cells with high copy number variation and enriched stem-like features. Functionally, *RAB30-DT* expression correlates with increased TMB, *TP53* mutation, genomic instability, and poor patient survival. Experimental validation demonstrated that *RAB30-DT* promotes tumor cell proliferation, invasion, migration, and colony and tumorsphere formation, highlighting its role in regulating CSC-like phenotypes and supporting HCC tumorigenicity. We also recognize the limitations of our current experiments on *RAB30-DT* and plan to perform in vivo limiting dilution assays in future studies to more accurately assess its effect on CSC frequency at the single-cell level.

Mechanistically, *RAB30-DT* drives extensive AS alterations, particularly in genes governing cell cycle and embryonic development. Among its targets, *CDCA7*—a chromatin remodeling gene involved in DNA methylation [46, 54, 55], embryonic stem cell maintenance [56], and gemcitabine resistance [48]—was identified as a key effector. While total *CDCA7* expression remained largely unchanged upon *RAB30-DT* knockdown, *CDCA7* variant 2 was selectively downregulated. This variant was found to be essential for maintaining CSC-like traits in HCC. These findings suggest a novel RAB30-DT–CDCA7 splicing axis underlying tumor stemness. Whether *CDCA7* variant 2 modulates chromatin dynamics or epigenetic reprogramming warrants further investigation. Elucidating this mechanism may uncover novel epigenetic vulnerabilities for targeting CSC-driven progression in HCC.

SRPK1 is a pivotal splicing regulatory kinase that phosphorylates serine/arginine-rich proteins to modulate their activity and localization. In HCC, elevated SRPK1 expression has been linked to disease progression and poor survival outcomes [57]. Overexpression of SRPK1 has also been documented in multiple cancers and is closely associated with tumor progression and poor prognosis [58, 59]. SRPK1 has been implicated in promoting cell proliferation [60], apoptosis [61], metastasis [62–64], angiogenesis [65–67], metabolic reprogramming [68], and resistance to chemotherapy [69, 70] and immunotherapy [71]. For example, MicroRNAs have been shown to suppress SRPK1 expression and inhibit HCC metastasis [62–64]. However, its role in governing CSC traits and stemness-related AS programs remains largely unexplored. Additionally, the regulatory mechanisms controlling SRPK1 function—beyond microRNA-mediated repression —have not been elucidated, especially with regard to lncRNA regulation.

In this study, our further mechanistic analysis revealed that *RAB30-DT* directly interacts with SRPK1, as confirmed by RNA pull-down, FISH, and immunofluorescence assays. Additionally, *RAB30-DT* enhances SRPK1 expression, protein stability, and nucleic translocation, thereby directing downstream splicing programs, including those of *CDCA7*. Our findings reveal that *RAB30-DT* may serve as a molecular scaffold, regulating SRPK1 activity and modulating SRPK1-mediated alternative splicing in favor of tumor-promoting isoforms. Upstream, we identified CREB1 as a transcriptional activator of *RAB30-DT*, supported by ChIP–qPCR, luciferase reporter, and cell functional assays. As CREB1 is a well-known oncogenic transcription factor involved in cancer progression and treatment resistance [51, 72–74], its regulation of *RAB30-DT* underscores a broader oncogenic network. Together, these results delineate a novel CREB1–RAB30-DT–SRPK1–CDCA7 regulatory axis that orchestrates CSC-like phenotypes via coordinated transcriptional and post-transcriptional mechanisms.

From a translational perspective, our drug sensitivity analysis revealed that high levels of *CREB1*, *RAB30-DT*, and *SRPK1*, along with elevated stemness features, were linked to reduced sensitivity to agents such as dasatinib and selumetinib. Functionally, *RAB30-DT* overexpression conferred drug resistance, which was reversed by *SRPK1* knockdown, highlighting *SRPK1* as a key mediator. Conversely, tumors with high *RAB30-DT* levels showed increased sensitivity to agents such as daporinad and belinostat—a vulnerability that was abolished upon *SRPK1* knockdown. These findings suggest that the CREB1–RAB30-DT–SRPK1 axis modulates not only CSC traits but also therapeutic response, providing a rationale for stratified treatment strategies. Potential therapeutic avenues include small-molecule inhibitors targeting CREB1 or SRPK1 and RNA-based therapies against *RAB30-DT*.

In summary, our study identifies *RAB30-DT* as a central regulator of splicing dysregulation and CSC-like phenotypes in HCC, acting through SRPK1 interaction and under CREB1 transcriptional control. This novel lncRNA-driven axis represents both a mechanistic insight into CSC regulation and a promising therapeutic vulnerability. Future work should aim to validate this pathway in larger patient cohorts, elucidate the epigenetic impact of *CDCA7* variant 2, and assess the efficacy of axis-targeted interventions in preclinical and clinical settings.

## Supplementary Information

Below is the link to the electronic supplementary material.

Supplementary Material 1

Supplementary Material 2

Supplementary Material 3

Supplementary Material 4

## Data Availability

The RNA sequencing data supporting the conclusions of this study have been deposited in the NCBI Gene Expression Omnibus (GEO) under accession number GSE298873 (https://www.ncbi.nlm.nih.gov/geo/query/acc.cgi?acc=GSE298873). The protein mass spectrometry data from the RNA pulldown experiments are accessible through the iProX database under project ID IPX0012124000 (https://www.iprox.cn/page/project.html?id=IPX0012124000).

## Abbreviations

A3SS: Alternative 3’ splice sites
A5SS: Alternative 5’ splice sites
AS: Alternative splicing
ChIP: Chromatin immunoprecipitatio
CNV: Copy number variation
CSCs: Cancer stem cells
HCC: Hepatocellular carcinoma
LncRNAs: Long noncoding RNAs
mRNAsi: MRNA stemness index
MS: Mass spectrometry
MXE: Mutually exclusive exons
qPCR: Quantitative PCR
RI: Retained introns
ROC: Receiver operating characteristic
scRNA-SEQ: Single-cell RNA-SEQ
SE: Skipped exons
SNV: Single nucleotide variation
SRPK1: Serine–arginine protein kinase 1
TMB: Tumor mutation burden
