# Supplementary material for: A novel lncRNA-mediated signaling axis governs cancer stemness and splicing reprogramming in hepatocellular carcinoma with therapeutic potential
Source: J Exp Clin Cancer Res. 2025 Oct 9;44:287. doi: 10.1186/s13046-025-03546-w (PMC12512555; doi:10.1186/s13046-025-03546-w)
Supplement: Supplementary file 1 — Supplementary Material 1 [file 13046_2025_3546_MOESM1_ESM.pdf]

Supplementary Figures

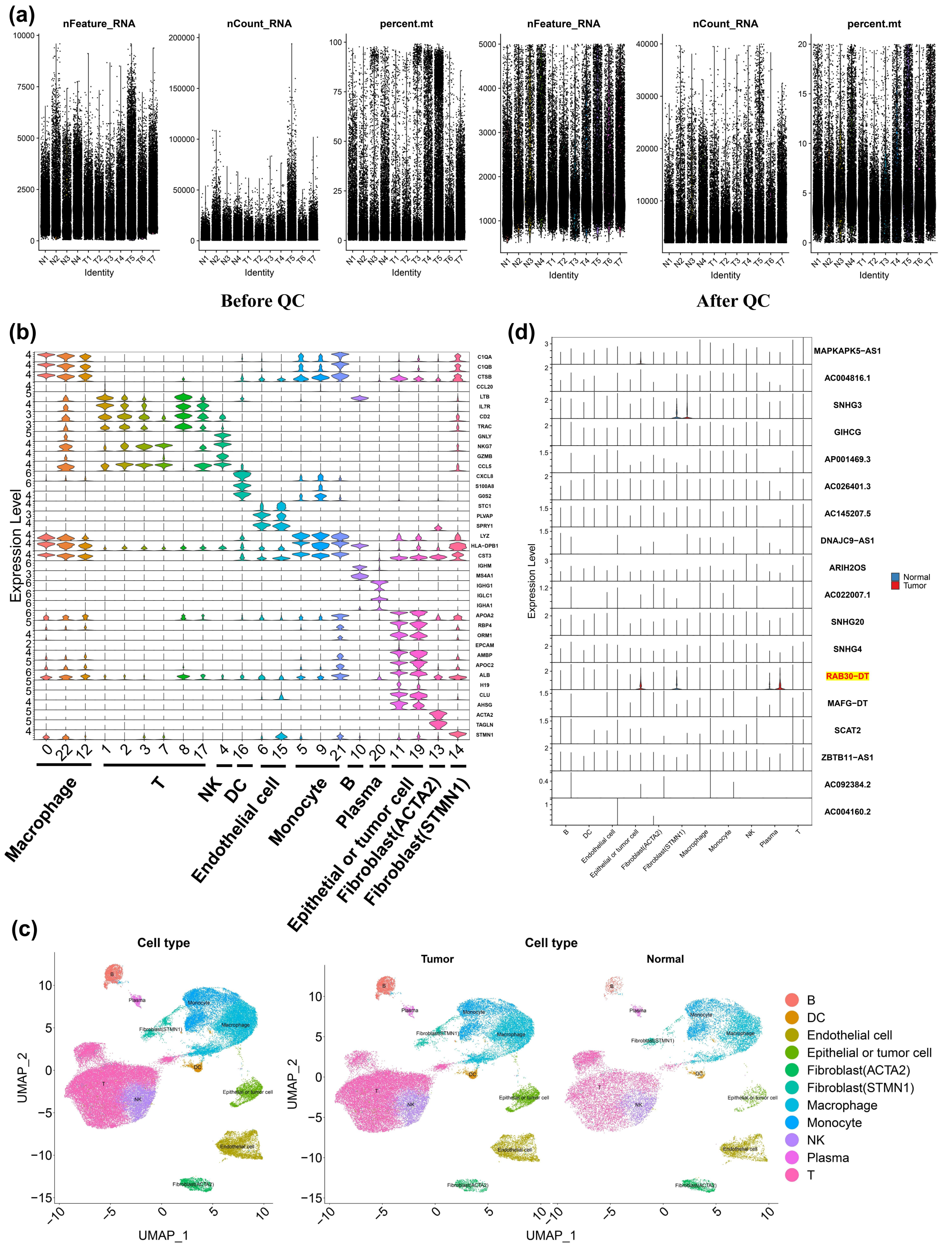

Supplementary Fig. 1. Quality control and analysis of the scRNA-SEQ dataset GSE202642. (a) Data quality control metrics, including the number of genes detected per cell, total counts, and mitochondrial gene content. **(b)** Cell type annotation based on canonical marker gene expression. **(c)** UMAP plot displaying distinct cell clusters identified in HCC tissue from the GSE202642

dataset. **(d)** Expression patterns of 19 splicing- and stemness-related lncRNAs across different cell types in HCC tissues.

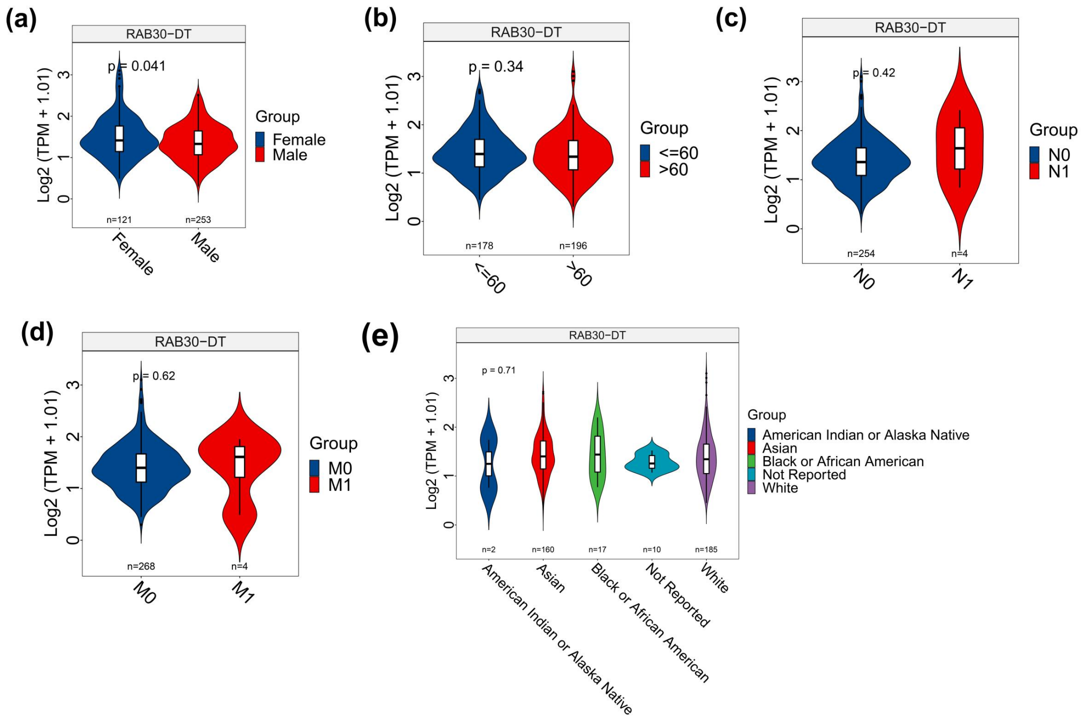

Supplementary Fig. 2. Association of LncRNA *RAB30-DT* expression with clinical characteristics in HCC patients. (a, b) *RAB30-DT* expression differs significantly between male and female patients but shows no association with age. **(c, d)** No significant correlation is observed between *RAB30-DT* expression and metastasis status or lymph node involvement. **(e)** *RAB30-DT* expression does not differ significantly among different racial groups.

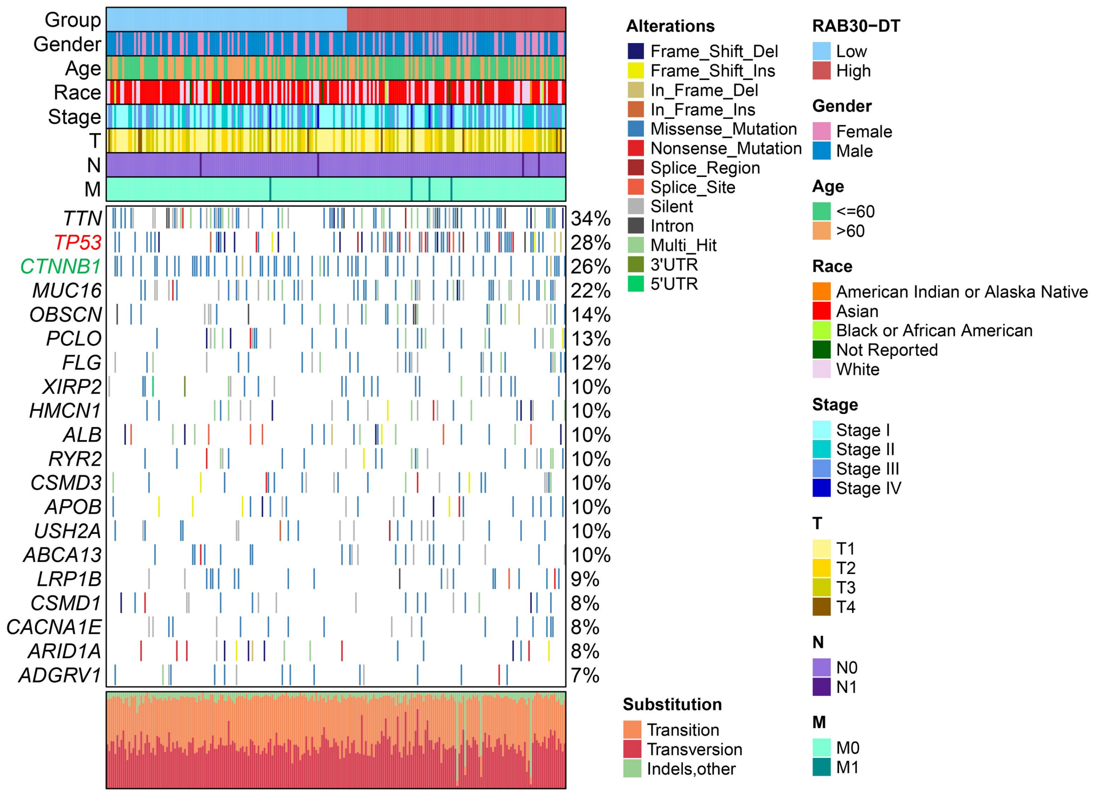

Supplementary Fig. 3. Waterfall plot of somatic mutations in HCC patients with high versus low lncRNA *RAB30-DT* expression from TCGA–LIHC data analysis.

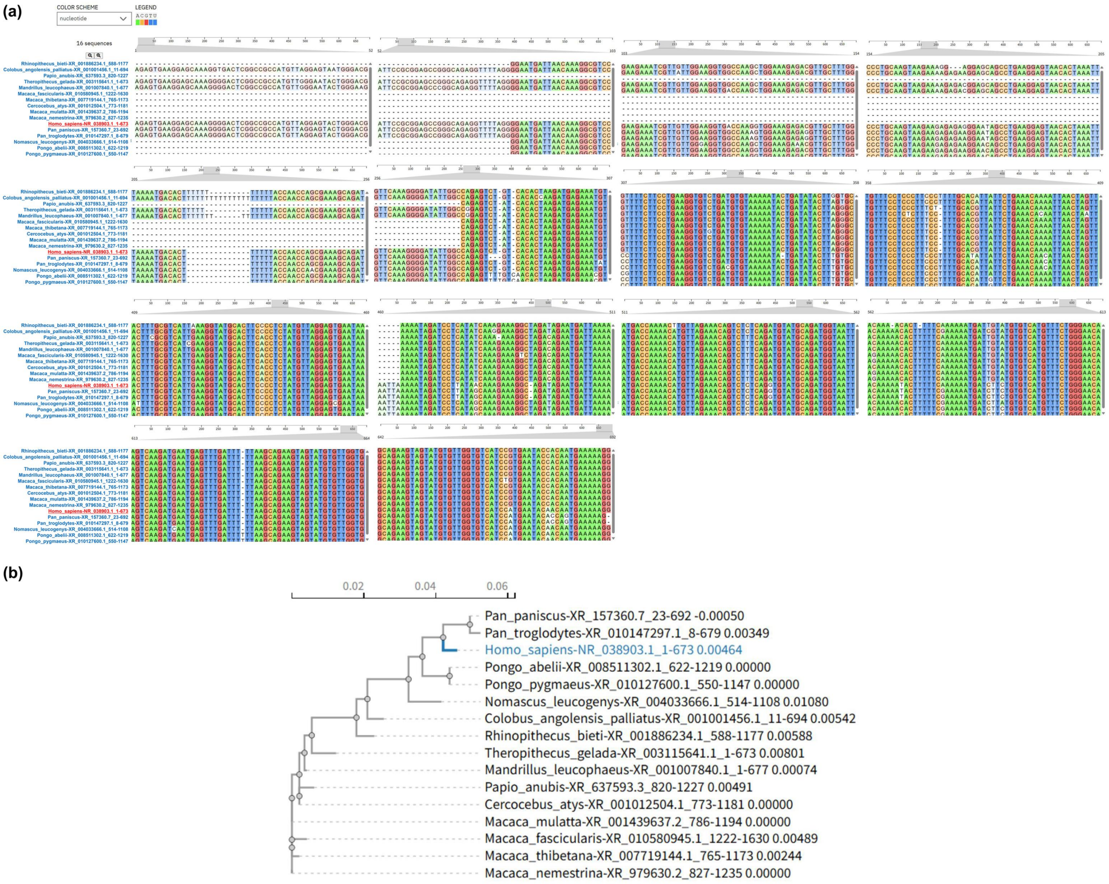

Supplementary Fig. 4. LncRNA *RAB30-DT* exhibits high sequence conservation among primates. (a) Multiple sequence alignment of human *RAB30-DT* with its orthologous transcripts in other primate species, generated using Clustal Omega. **(b)** Phylogenetic tree constructed based on *RAB30-DT* and its orthologous transcript sequences across primates, using Clustal Omega.

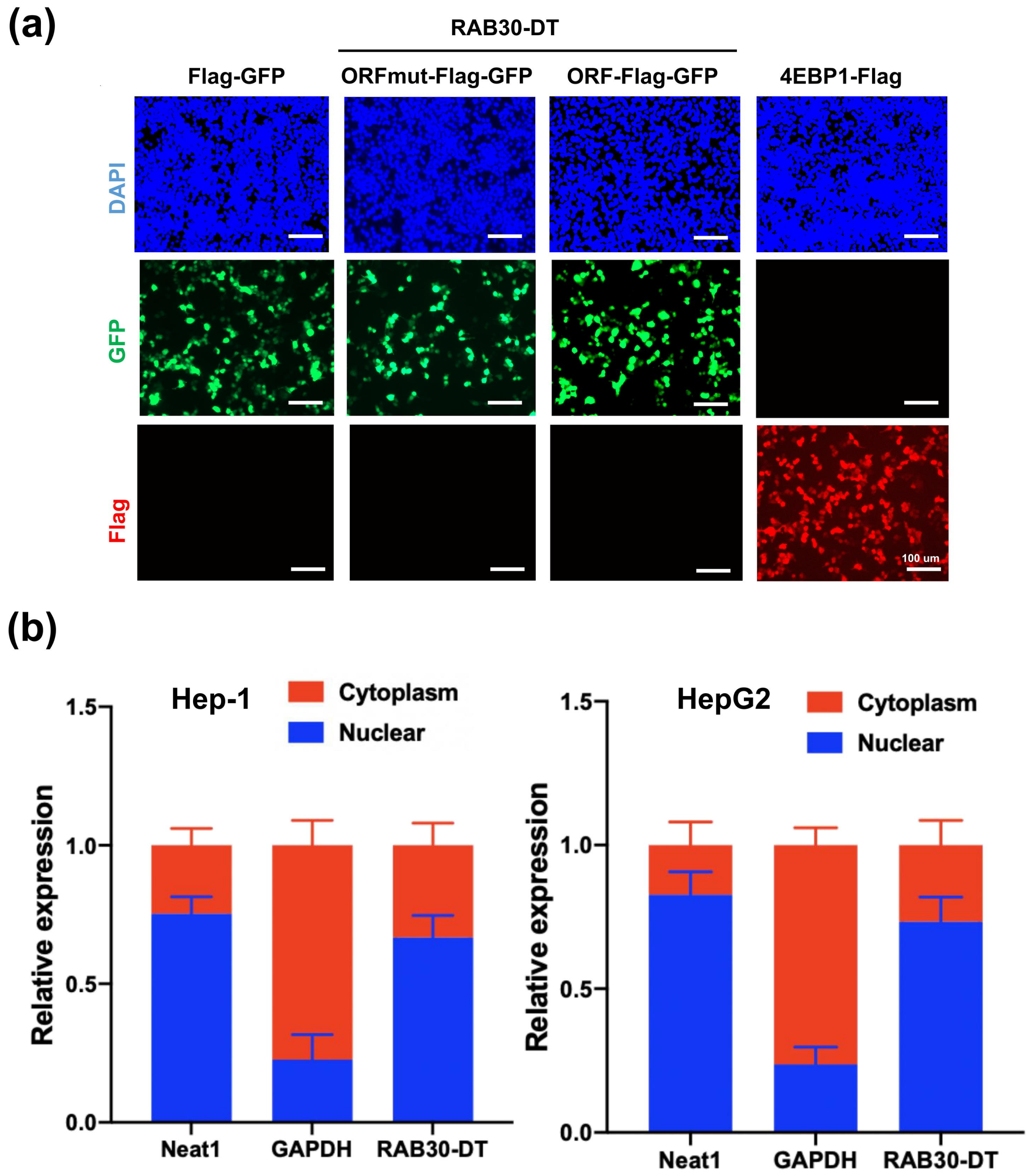

Supplementary Fig. 5. Characterization of the coding potential and subcellular localization of lncRNA *RAB30-DT*. (a) Immunofluorescence analysis indicates that *RAB30-DT* does not encode a detectable protein. **(b)** Nucleus-cytoplasm fractionation followed by qPCR reveals that *RAB30-DT* is predominantly localized in the nucleus.

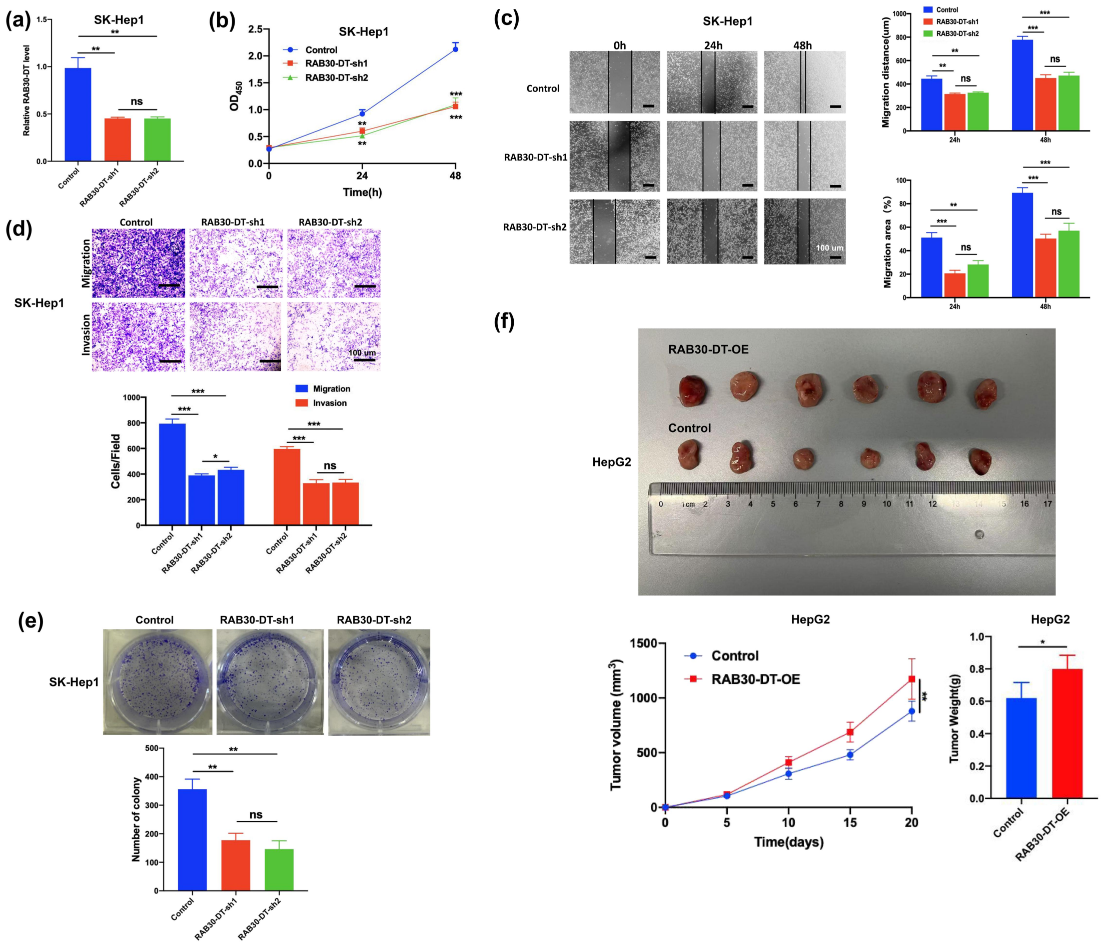

Supplementary Fig. 6. Functional effects of *RAB30-DT* in SK-Hep-1 cells and *in vivo* tumorigenesis. (a) qPCR validation of *RAB30-DT* knockdown efficiency in SK-Hep-1 cells. *RAB30-DT* knockdown suppresses **(b)** proliferation, **(c)** migration, **(d)** invasion, and **(e)** colony formation in SK-Hep-1 cells, whereas *RAB30-DT* overexpression promotes xenograft tumor growth of HepG2 cells in nude mice **(f).**

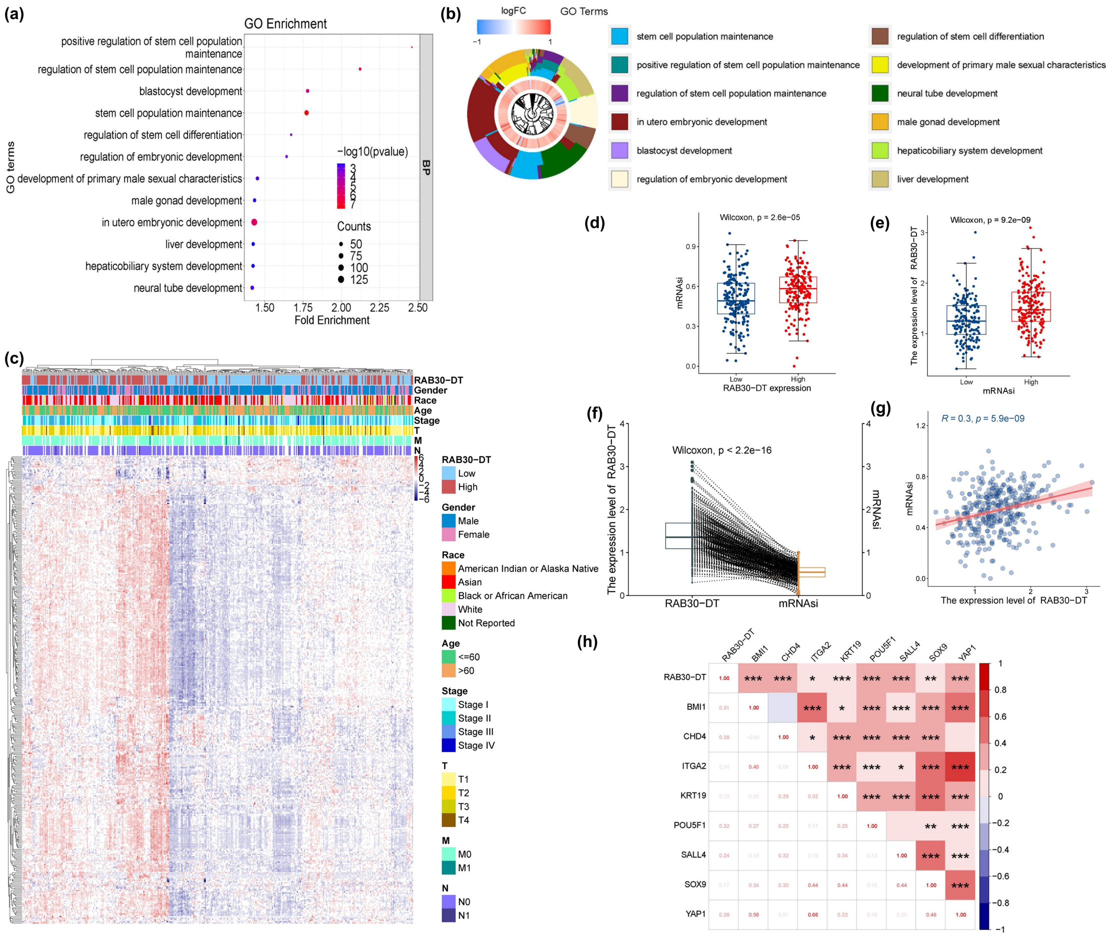

Supplementary Fig. 7. LncRNA *RAB30-DT* expression is positively associated with tumor stemness. (a–b) Gene Ontology analysis of differentially expressed genes (DEGs) between high and low *RAB30-DT* expression groups in TCGA–LIHC. **(c)** Heatmap of DEGs enriched in stemness-related pathways between high and low *RAB30-DT* expression groups. **(d–g)** *RAB30-DT* expression is significantly positively correlated with mRNAsi scores. **(h)** Positive correlation between *RAB30-DT* expression and cancer stemness–related genes.

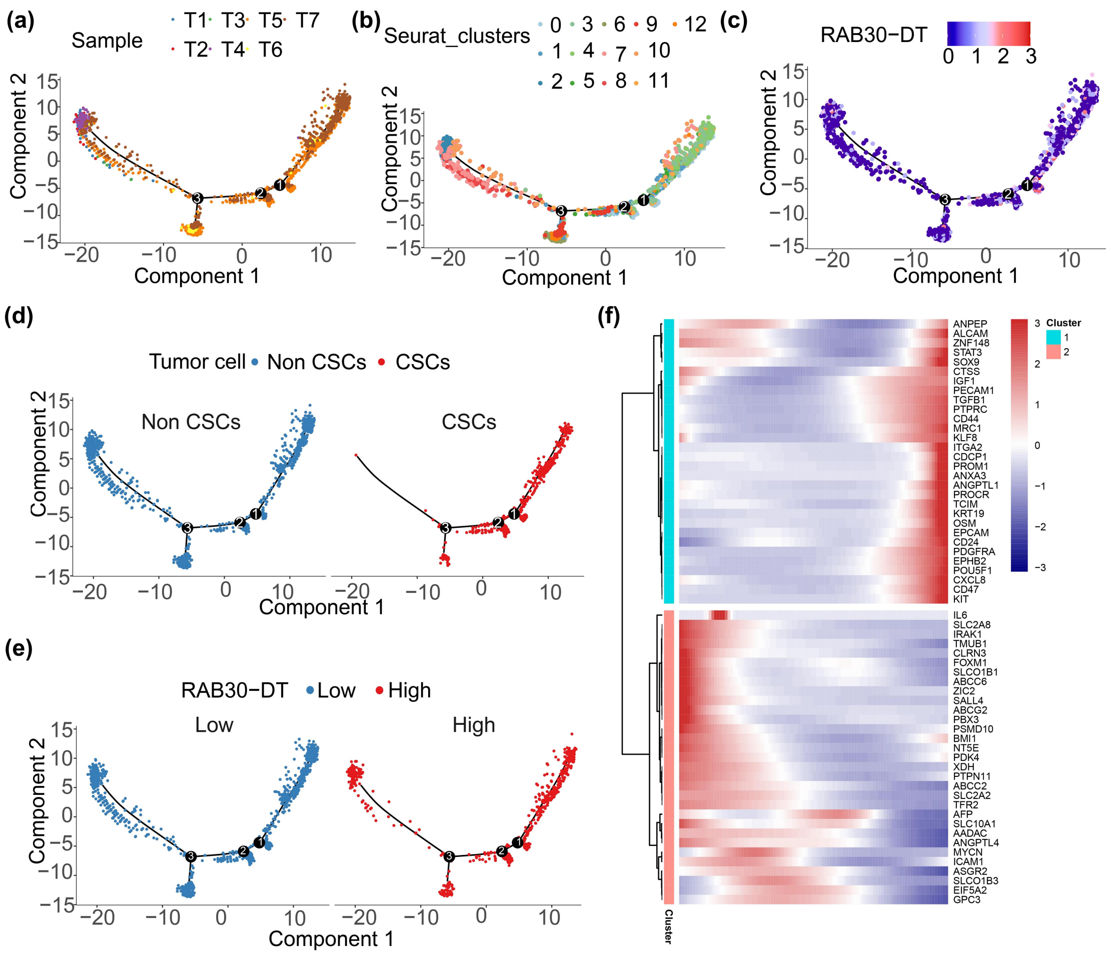

Supplementary Fig. 8. Pseudotime trajectory analysis reveals that high lncRNA *RAB30-DT* expression occurs in early pseudotime stages and is associated with elevated stemness potential in malignant cells. (a) Pseudotime trajectory of tumor epithelial cells across different HCC patients, showing inferred developmental progression. **(b)** Pseudotime trajectory of HCC cells colored by distinct cell clusters. **(c)** Pseudotime trajectory highlighting HCC cells with different levels of *RAB30-DT* expression. **(d)** Pseudotime distribution of cancer stem-like cells (CSCs) and non–CSCs, indicating their temporal positioning. **(e)** Comparison of pseudotime trajectories between *RAB30-DT* high– and low–expressing HCC cells. **(f)** Branched Expression Analysis Modeling identifies genes differentially expressed at the trajectory branching point, potentially involved in cell fate decisions.

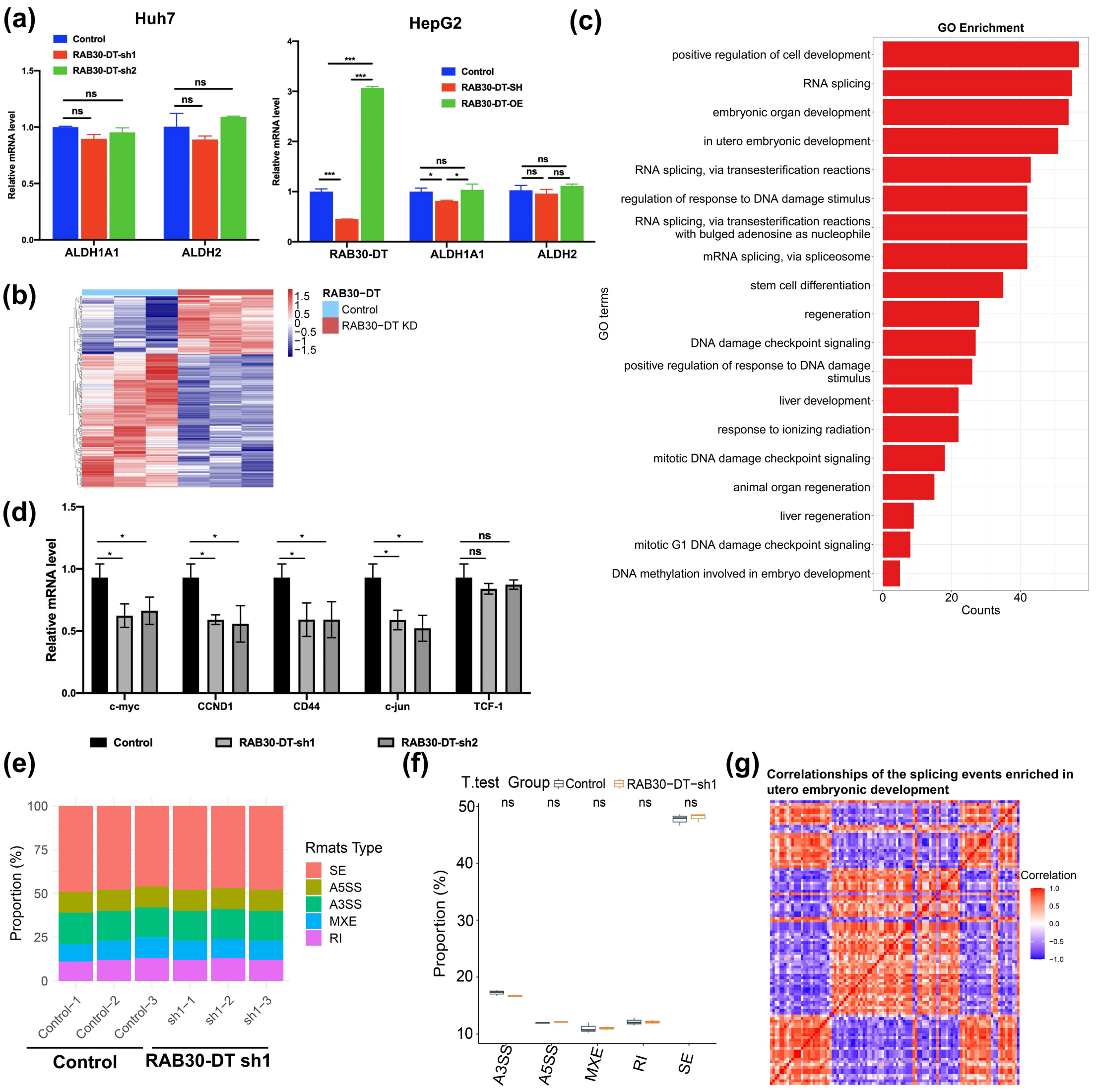

Supplementary Fig. 9. RNA-SEQ and alternative splicing analysis reveal the impact of lncRNA *RAB30-DT* knockdown in HCC cells. (a) qPCR analysis of *ALDH1A1* and *ALDH2* expression following *RAB30-DT* knockdown or overexpression in Huh7 and HepG2 cells. **(b)** Heatmap showing global gene expression changes in *RAB30-DT* knockdown versus control cells. **(c)** GO analysis of DEGs in *RAB30-DT* knockdown cells. **(d)** qPCR validation of DEGs in stemness-related pathways upon *RAB30-DT* knockdown. **(e–f)** Proportional distribution of alternative splicing (AS) event types between *RAB30-DT* knockdown and control cells. **(g)** Co-occurrence network of differentially spliced genes between *RAB30-DT* knockdown and control groups.

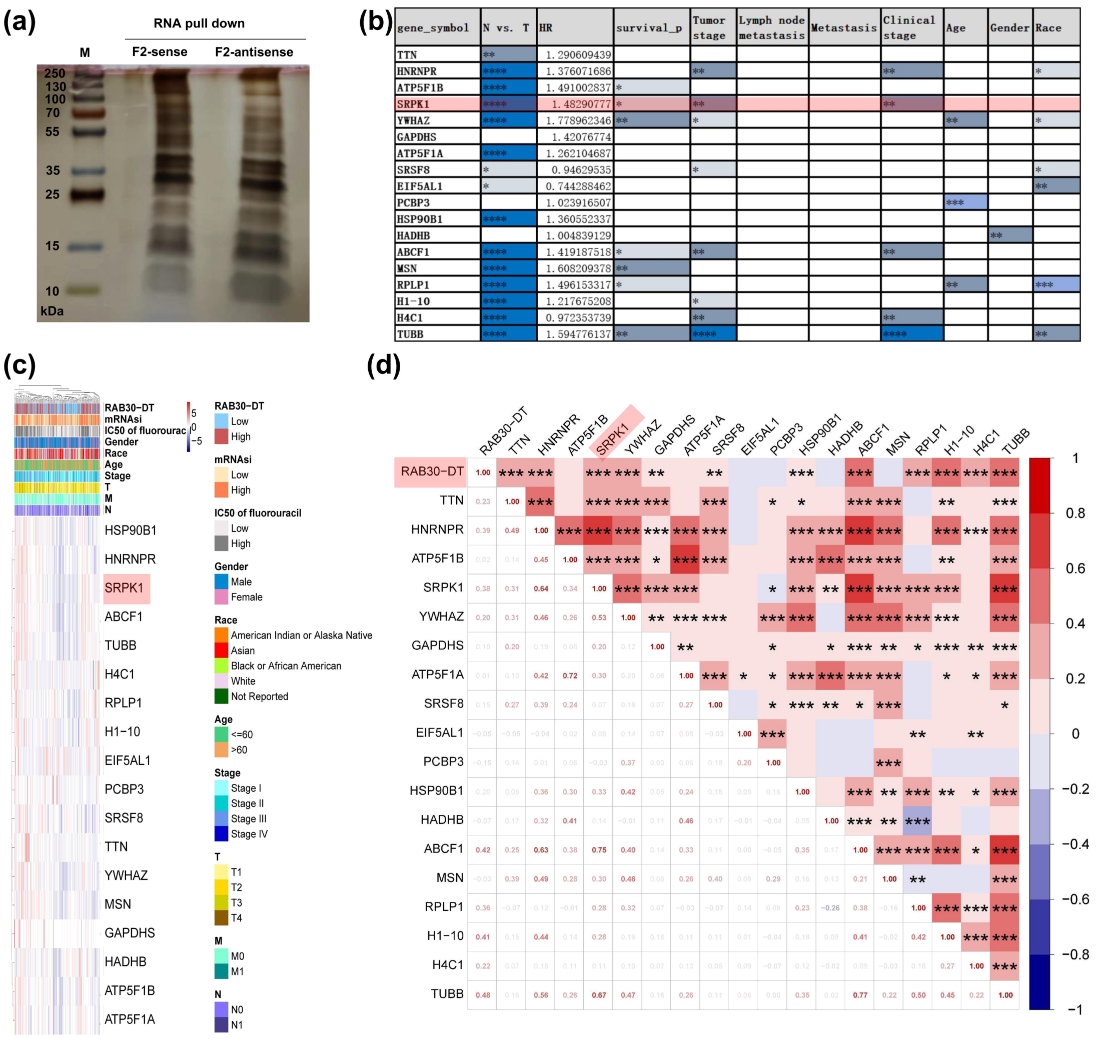

gene_symbol	N vs. T	HR	survival_p	Tumor stage	Lymph node metastasis	Metastasis	Clinical stage	Age	Gender	Race
TTN	**	1.290609439								
HNRNPR	****	1.376071686		**			**			*
ATP5F1B	****	1.491002837	*							
SRPK1	****	1.48290777	*	**			**			
YWHAZ	****	1.778962346	**	*				**		*
GAPDHS		1.42076774								
ATP5F1A	****	1.262104687								
SRSF8	*	0.94629535		*						*
EIF5AL1	*	0.744288462								**
PCBP3		1.023916507						***		
HSP90B1	****	1.360552337								
HADHB		1.004839129							**	
ABCF1	****	1.419187518	*	**			**			
MSN	****	1.608209378	**							
RPLP1	****	1.496153317	*					**		***
H1−10	****	1.217675208		*						
H4C1	****	0.972353739		**			**			
TUBB	****	1.594776137	**	****			****			**

Supplementary Fig. 10. RNA pull–down proteomics identifies binding partners of lncRNA *RAB30-DT*. (**a**) Silver staining following RNA pull–down reveals differential proteins pulled down by the sense and antisense strands of *RAB30-DT*. **(b)** TCGA–LIHC analysis of RNA pull–down proteins, followed by mass spectrometry, identifies specific proteins whose gene expression correlates with HCC development and prognosis. **(c)** TCGA–LIHC analysis shows that the expression of RNA pull–down identified proteins is obviously elevated in HCC patients with high *RAB30-DT* expression. **(d)** The gene expression of RNA pull–down proteins correlate significantly with *RAB30-DT* expression, as demonstrated by mass spectrometry analysis.

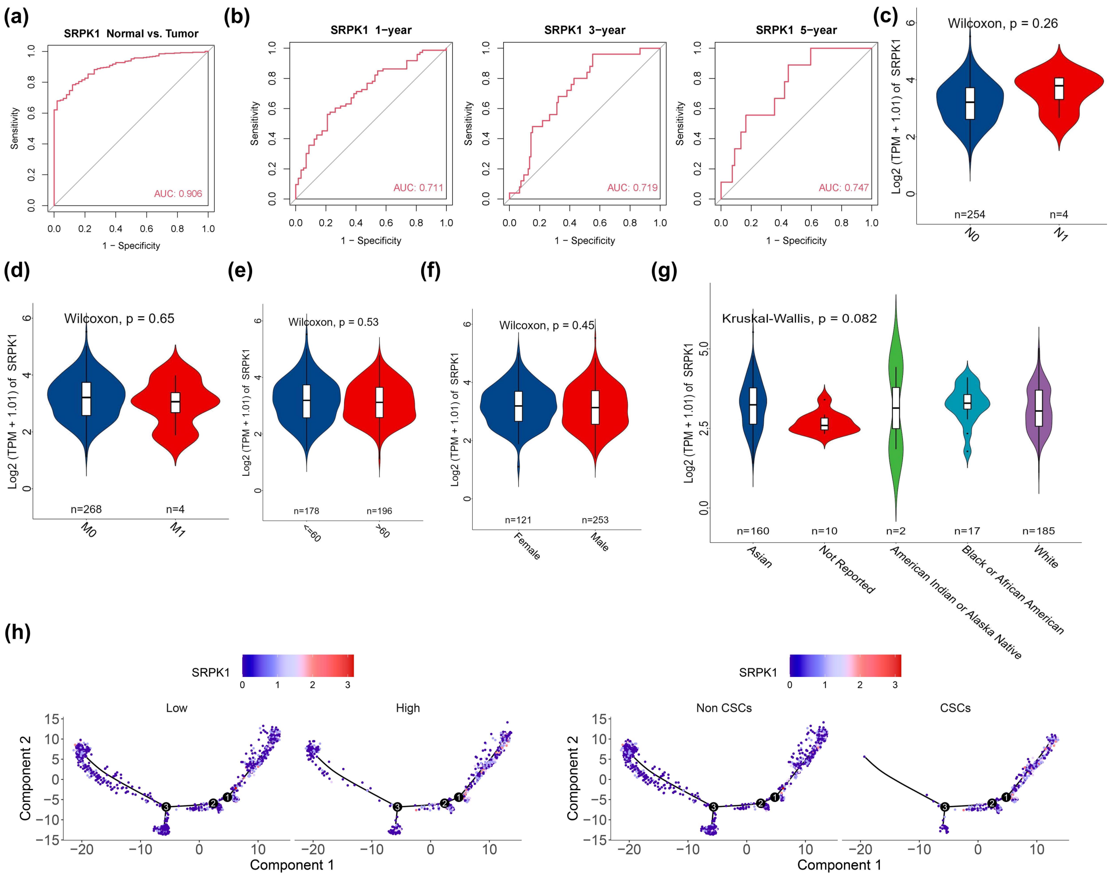

Supplementary Fig. 11. Clinical relevance of *SRPK1* in HCC. (a) *SRPK1* is significantly upregulated in HCC tissues (TCGA–LIHC). **(b)** *SRPK1* expression predicts 1-, 3-, and 5-year survival outcomes in HCC patients. **(c–g)** Correlations of *SRPK1* expression with lymph node metastasis, distant metastasis, age, gender, and race. **(h)** Pseudotime analysis links *SRPK1* expression with malignant cell differentiation states.

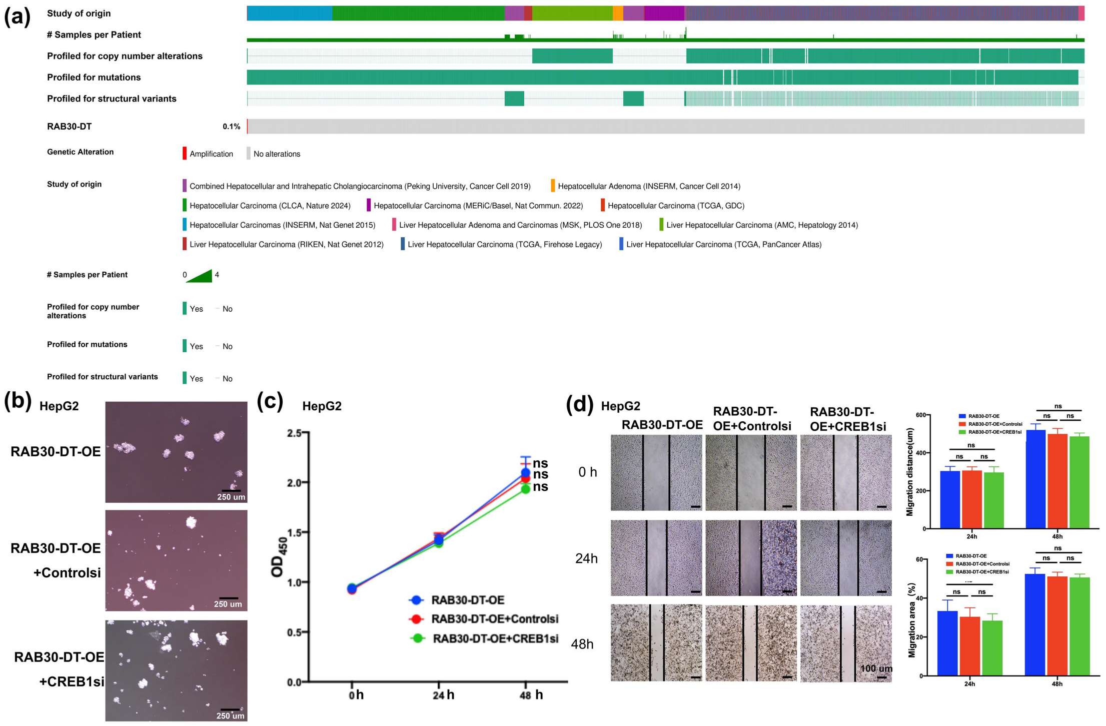

Supplementary Fig. 12. *RAB30-DT* functions downstream of *CREB1* to regulate tumor stemness and malignancy in HCC. (a) cBioPortal analysis reveals that genomic alterations of the lncRNA *RAB30-DT* are rare in hepatocellular carcinoma (HCC). **(b–d)** Functional assays assessing the impact of *CREB1* knockdown in HepG2 cells overexpressing *RAB30-DT* on tumor stemness (b), proliferation (c), and migration (d).
