# Supplementary material for: A novel lncRNA-mediated signaling axis governs cancer stemness and splicing reprogramming in hepatocellular carcinoma with therapeutic potential
Source: J Exp Clin Cancer Res. 2025 Oct 9;44:287. doi: 10.1186/s13046-025-03546-w (PMC12512555; doi:10.1186/s13046-025-03546-w)
Supplement: Supplementary file 3 — Supplementary Material 3 [file 13046_2025_3546_MOESM3_ESM.doc]

**Table S2. Predicted Transcription Factors Binding to the lncRNA RAB30-DT Promoter and Their Clinical Relevance in HCC**

| **TFs** | **Normal vs. Tumor** | **Survival** | **Clinical satge (I vs. II vs. III&IV)** | **T satge (T1 vs. 2 vs. 34)** | **N stage (N0 vs. N1)** | **M stage (M0 vs. M1)** | **Correlated with RAB30-DT** | **TFs binding Score of RAB30-DT** |
| --- | --- | --- | --- | --- | --- | --- | --- | --- |
| THAP11 | ***（Up） | *（Poor） | - | - | - | - | ****（Positive） | 717 |
| RREB1 | ***（Up） | - | - | - | - | - | ****（Positive） | 714 |
| JUN | ***（Down） | - | - | - | - | - | - | 667 |
| OVOL1 | - | - | - | - | - | - | - | 659 |
| JUND | *（Down） | - | - | - | - | - | *（Positive） | 648 |
| ZNF76 | ***（Up） | *（Poor） | - | - | - | - | ****（Positive） | 612 |
| OVOL2 | ***（Down） | - | - | - | - | - | *（Positive） | 608 |
| ATF2 | ***（Up） | - | *（Up） | *（Up） | - | - | ****（Positive） | 607 |
| POU6F2 | ***（Down） | - | - | - | - | - | *（Positive） | 602 |
| STAT2 | ***（Up） | - | - | - | - | *（Down） | ****（Positive） | 597 |
| **CREB1** | *****（Up）** | ****（Poor）** | ****（Up）** | ***（Up）** | **-** | **-** | ******（Positive）** | **584** |
| POU6F1 | ***（Up） | - | - | - | - | *（Down） | - | 571 |
| PRDM9 | ***（Up） | ***（Poor） | *（Up） | - | - | - | ****（Positive） | 569 |
| ZIC1 | - | - | - | - | - | - | *（Positive） | 569 |
| ZNF143 | ***（Up） | *（Poor） | *（Up） | *（Up） | - | - | ****（Positive） | 568 |
| MAFK | - | - | - | - | - | - | - | 567 |
| KLF9 | ***（Down） | *（Better） | - | - | - | - | - | 561 |
| IRF1 | *（Up） | - | - | - | - | - | *（Positive） | 554 |
| FOXD2 | ***（Up） | **（Poor） | - | * | - | - | **（Positive） | 553 |
| ZNF708 | ***（Up） | - | - | - | - | - | ****（Positive） | 552 |
| FOXE1 | - | - | ***（Up） | ***（Up） | - | - | - | 550 |
| RORB | ***（Up） | - | - | - | - | - | *（Negative） | 550 |
| PRDM1 | - | - | - | - | - | - | - | 536 |
| PATZ1 | ***（Up） | *（Poor） | - | - | - | - | ****（Positive） | 531 |
| KLF4 | ***（Down） | - | - | *（Up） | - | - | - | 530 |
| KLF16 | ***（Up） | - | - | - | - | - | - | 527 |
| SP8 | ***（Up） | - | - | - | - | - | ****（Positive） | 526 |
| SMAD2 | ***（Up） | - | **（Up） | **（Up） | *（Up） | - | ****（Positive） | 524 |
| PBX2 | ***（Up） | - | - | - | - | - | ****（Positive） | 523 |
| ZNF675 | ***（Up） | - | - | - | *（Up） | *（Down） | ****（Positive） | 522 |

*: *p*≤0.05; **: *p*≤0.01; ***: *p*≤0.001; ****: *p*≤0.0001; -:No significance.
